# Assessment of Nontarget Small Mammal Occupancy Using Broadly Designed Camera Arrays

**DOI:** 10.1002/ece3.73834

**Published:** 2026-06-19

**Authors:** Ashley M. Olah, Laken S. Ganoe, Christopher J. Hickling, Theint Thandar Bol, Donald Ruggieri, Amy Mayer, Kathleen A. Carroll

**Affiliations:** ^1^ Quest Lab, Department of Natural Resources Science University of Rhode Island Kingston Rhode Island USA; ^2^ Meyerson Lab, Department of Natural Resources Science University of Rhode Island Kingston Rhode Island USA

**Keywords:** bycatch, camera traps, cottontail, occupancy dynamics, rodent, squirrel

## Abstract

The use of camera traps has become a mainstay method in ecological research and monitoring, particularly for rare, cryptic, or remote species. While many camera trap study designs focus on a single species, many nontarget species, or “bycatch,” are captured as well. This bycatch can be leveraged to address questions beyond the scope of the original study designs. We aimed to assess the distributions and trends in the occurrence of nontarget species captured by an existing camera trap study initially designed to monitor mesocarnivores in Rhode Island. Using dynamic occupancy models in a Bayesian framework, we modeled the seasonal occupancy of eastern gray squirrels ( *Sciurus carolinensis* ), red squirrels (*Tamiasciurus hudsonicus*), eastern chipmunks ( *Tamias striatus* ), cottontails (*Sylvilagus* spp.), and small ground‐dwelling rodents (spp. *undistinguished*) for each year from 2019 to 2023. We found that gray squirrel occupancy was mainly driven by annual variation and net primary productivity, a proxy for food availability. Red squirrel dynamics were influenced by the amount of coniferous forest around a site, seasonal variation, and human modification. Cottontail dynamics were positively related to the presence of young forests and negatively to human modification. Eastern chipmunk dynamics were related to seasonal timing, forest recovery after spongy moth ( *Lymantria dispar* ) damage, and precipitation. Small rodent dynamics were driven by forest structure, age, and local site features. We found that occupancy probabilities of gray squirrels, eastern chipmunks, cottontails, and small rodents increased from 2019 to 2023, and red squirrel occupancy probability showed a minor decline across Rhode Island. Our findings indicate that data on nontarget species captured via existing camera trap studies can be utilized to provide important insights about species that may not otherwise be assessed, ecosystem health, and provide potential predictors for use in analyses of target species.

## Graphical abstract

Camera traps are a mainstay method in ecological research and monitoring, often focusing on a single species; however, many nontarget species are captured as well. We aimed to assess the distributions and trends in the occurrence of nontarget species captured by an existing camera trap study initially designed to monitor mesocarnivores in Rhode Island, and found all species' occupancy increased over time. Our findings indicate that data on nontarget species captured via existing camera trap studies can be utilized to provide important insights about species that may not otherwise be assessed.

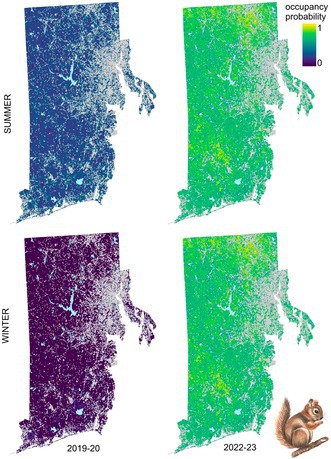

## 1 Introduction

Using remote cameras, or “camera trapping,” has become increasingly common over the past decades, revolutionizing ecological research and monitoring (Fisher 2025; Rovero and Zimmermann 2016). In particular, cameras have proven an important alternative for monitoring rare, cryptic, or remote species (Kelly 2008; Vine et al. 2009) when other survey methods (e.g., manned aerial surveys, transects) are not possible due to lack of resources (i.e., funds, time; De Bondi et al. 2010; Rowcliffe et al. 2008) or other limitations (e.g., access to airspace, researcher safety concerns). While camera arrays are increasingly designed to target multiple species (e.g., Carroll et al. 2025; Ganoe et al. 2024), many designs focus on single species, particularly when such species are rare or elusive (e.g., Kortello et al. 2024; Shannon et al. 2023). More recently, researchers have begun leveraging camera data to address questions beyond the scope of the original study design, exploring the utility of cameras for nontarget species (sensu Hofmeester et al. 2020; Williams et al. 2021). In this way, remote camera data can be leveraged to capture a wide array of species, many of which might not be otherwise monitored, depending on several features of the target species(s) and study area or system. For example, camera trap bycatch has been used to evaluate the spread of sarcoptic mange in red foxes ( *Vulpes vulpes* ) (Pisano et al. 2019), generate range‐wide population estimates of sun bears ( *Helarctos malayanus* ) (Scotson et al. 2017), improve knowledge of Asian tapir ( *Tapirus indicus* ) ecology and abundance (Petersen et al. 2026), predict occupancy of brown hyaena ( *Parahyaena brunnea* ) (Williams et al. 2021) and pangolins (Pholidota: Manidae) (Khwaja et al. 2019) to aid conservation, and investigate co‐occurrence, niche partitioning, activity patterns, and species interactions (Caravaggi et al. 2017; Sweitzer and Furnas 2016).

The capacity for a camera array to capture a vast assortment of species (including both the target and nontarget species) relies on a foundation of robust study design. Several core components of robust camera array design include the number of cameras on the landscape, the scale at which cameras are spaced (the underlying sampling grid), and how camera placement is assigned (e.g., random, stratified random, etc.). The density (i.e., the number of cameras per area), which encompasses both the number and spacing of cameras on an underlying grid, may preclude strong inference for nontarget species if the grid and area are too large. For example, a 15 × 15 km wolverine‐targeted ( *Gulo gulo* ) grid, as used by Lukacs et al. (2020), is unlikely to support inference regarding species with much smaller home ranges (e.g., marten) without modification. However, for species with less extreme ranges (e.g., New England mesocarnivores), the spatial arrangement of cameras is more likely to capture sufficient naive occupancy for a broader range of species (e.g., Ganoe et al. 2024). In such instances, data on nontarget species, also called incidental or bycatch detections, can provide insights about species that might not otherwise be assessed. Such bycatch can include common species and those not harvested or extensively regulated. Yet, these data may provide important insights about ecosystem processes (e.g., prey food availability) that researchers are interested in quantifying as reflections of system health and potential predictors for target species analyses. For example, in the northeastern United States, the assessment of common rodent or rabbit species population trends could be used to inform management of both their habitat and their predators (i.e., mesocarnivores).

Eastern chipmunks ( *Tamias striatus* ), red squirrels (*Tamiasciurus hudsonicus*), eastern gray squirrels ( *Sciurus carolinensis* ), and cottontails (*Sylvilagus* spp.) are some of the small mammals commonly captured as bycatch in camera trap studies aimed at monitoring alternate taxa across the northeastern United States (Ganoe et al. 2024; Hofmeester et al. 2020; Mayer et al. 2025; Williams et al. 2021). These species are widely distributed throughout their native ranges, use a variety of different habitats (Kay et al. 2023), and are often considered indicators of ecosystem health due to their environmental sensitivity (Pearce and Venier 2005; Schmidt‐Nielsen 1984). Despite having a significant impact on ecosystem function and resiliency (e.g., prey items, invertebrate control, seed dispersal; Carey and Harrington 2001) and being economically important in many parts of the country (e.g., food, fur, ecotourism; Boland and Litvaitis 2008; Kays et al. 2017) they are often underrepresented in research (dos Santos et al. 2020), seemingly lacking charisma compared to larger mammals. Despite these species being underrepresented in research, they often have high naïve occupancy in camera trap studies and are predominantly diurnal (Boon et al. 2008; La Zerte and Kramer 2016) or crepuscular (Abu Baker et al. 2015), exhibiting peak activity during hours that would coincide with optimal camera trap operation.

Often, research using trail camera surveys for large mammals, particularly carnivores, fails to investigate the key driving factor of species distributions and trends: prey distribution and trends. In Rhode Island, USA, declines in key mesocarnivores have been documented, leading to speculation on the ties of these declines to distributions of prey species (Ganoe et al. 2024). In Rhode Island there is a strong gradient of wildland to urban land use, human disturbance in the form of development and paved roads, abundant water bodies, a temperate climate, and there have been drastic changes in the forest composition due to urbanization and an insect outbreak (Dumarevskaya and Parent 2025; Pasquarella et al. 2018). All of these factors can influence small mammal distributions, thus in turn impacting mesocarnivores. Here, our primary goals were to assess the distribution and trends in occurrence for nontarget species using an existing camera trap dataset initially designed to monitor mesocarnivores. We aimed to assess occupancy trends of the most commonly detected prey bycatch (e.g., gray squirrels, red squirrels, eastern chipmunks, cottontails, other ground‐dwelling rodents (spp. undistinguished, generally e.g., mice, voles, shrews, mole spp.; hereafter “small rodents”)) to understand what factors drive each species' distributions and to determine if species may be in decline. Generally, we expected that most species would be relatively generalist in nature, and occupancy would be driven by food availability, human modification, forest structure, and season. We expected spongy moth ( *Lymantria dispar* ) defoliation would alter small mammal occupancy due to a reduction in food resources (e.g., acorns), or changes in forest succession, tree species composition, forest age structure, or understory conditions which in turn alter habitat suitability (Gottschalk 1989; Barker Plotkin et al. 2025). However, we tailored predictions for each species based on its unique ecology (Table A1).

## 2 Methods

### 2.1 Study Area

**FIGURE 1:**
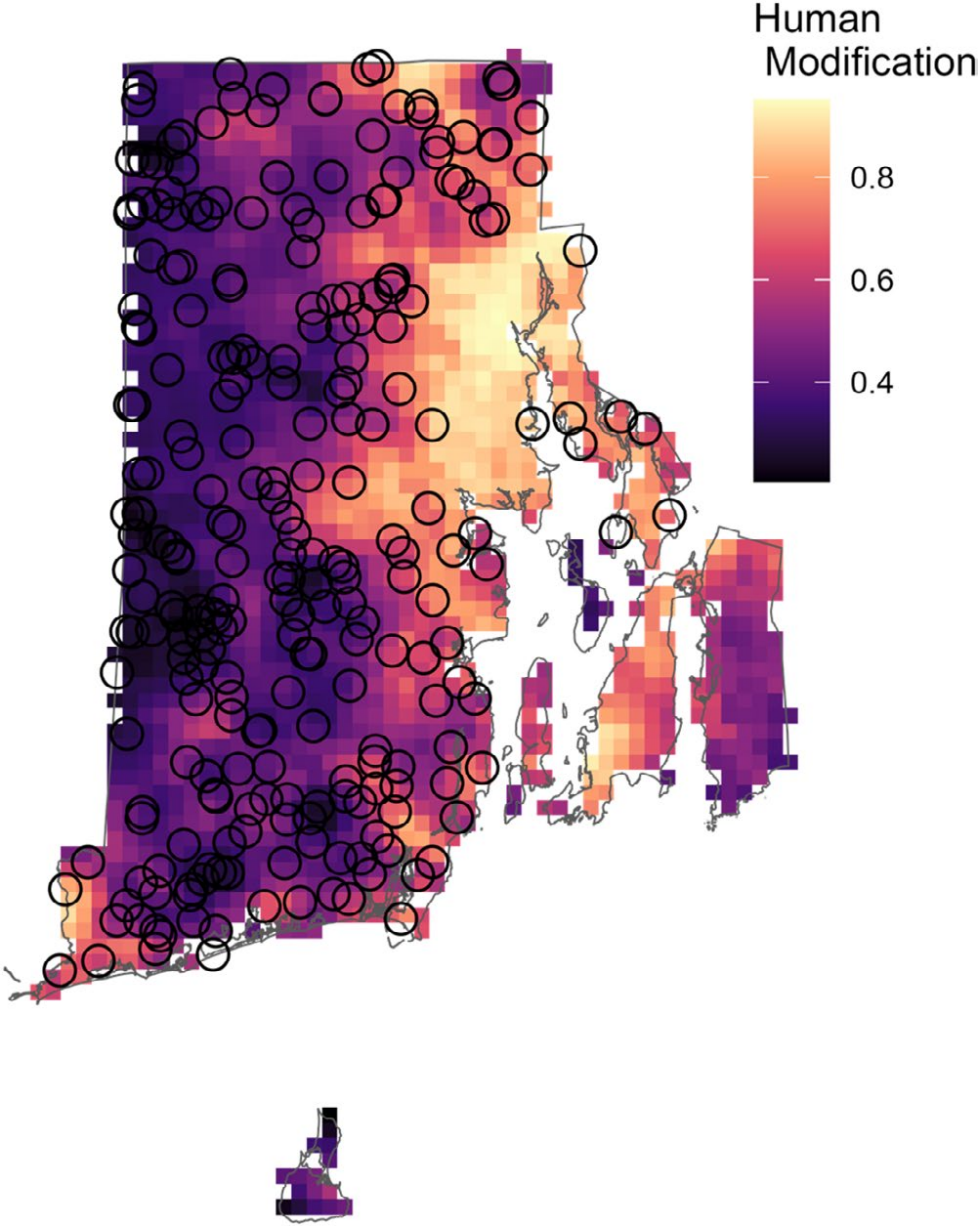
Study area map of camera trap locations across Rhode Island, USA from 2019–2023, and the global human modification map (Kennedy et al. 2019). Higher human modification values indicate more human modification of the environment.

We used data collected from a previous camera trapping study between 2019 and 2023 in Rhode Island (41.67° N, −71.61° W; Mayer et al. 2025; Figure 1) to examine rodent and cottontail occupancy trends. While the study was designed to detect mesocarnivore species, particularly bobcats ( *Lynx rufus* ) and fishers ( *Pekania pennanti* ), small mammals were also frequently detected on survey cameras. The small mammal community in Rhode Island is comprised primarily of squirrels (e.g., *Sciurus carolinensis* , *Tamiasciurus hudsonicus*, *Tamias striatus* ), cottontails (e.g., *Sylvilagus* sp.), and other small rodents (e.g., mice, voles, shrews, and mole species). While Rhode Island is densely populated (410 people/km^2^), most of the study area was forested (46.6%) or woody wetlands (11.6%). Additionally, many of the Rhode Island forests have at one point been farmed (Foster 1992; Lorimer 2001), and remain in agricultural use (8.9%). New England has a temperate climate, and the average annual temperatures in Rhode Island between 2019 and 2023 were 18.8°C in summer and 4.7°C in winter. Average precipitation in our study area ranged from 14.9″ of rain in summer to 25.8″ of snow in winter between 2019 and 2023. Much of Rhode Island's deciduous forests were defoliated and decimated by a larval spongy moth outbreak between 2015 and 2017 that has since altered forest successional states across our study area (Pasquarella et al. 2018).

### 2.2 Camera Data

Cameras were initially placed as part of a focal study on bobcats (2018–2020) and later fishers (2020–2023), primarily in forests and forested wetlands and avoiding trails, roads, and highly developed areas (Mayer et al. 2025; Figure 1). The average distance between camera sites was 1794 m (range: 135 m–7729 m). Two cameras (Browning Strike Force Pro XD, Browning, Morgan, UT, USA; or Bushnell Trophy Cam; Bushnell Outdoor Products, Overland Park, KS, USA) were placed per site, on average 55 m apart (range 20 m–130 m), in locations which would maximize detections of mesocarnivores, such as game trails, rock walls, fallen logs, and habitat edges. Cameras were placed 50 cm to 1 m above the ground, angled parallel to the ground, facing north, and vegetation in the field of view was trimmed. Cameras used red glow infrared flash for night photos, had average detection distances of 80 ft, and an average trigger speed of 0.15 s. Cameras took bursts of three photos when triggered by movement, with a 10 s delay between bursts. Scent lure (Caven's Gusto, Minnesota Trapline Products, Pennock, MN, USA) was applied 3–4 m in front of each camera, approximately 1–2 m above the ground, to increase mesocarnivore detections. From 2019–2023, two cameras were placed at each site 50 to 100 m apart, to maximize detections (Mayer et al. 2022). In each survey period, cameras were deployed for a minimum of 6 weeks each in winter and summer, at a maximum of 240 sites, over 12 survey periods (Table 1; Mayer et al. 2025). Cameras documented all species of terrestrial vertebrates within camera view at survey locations; however, we were interested in the data on gray squirrels, red squirrels, eastern chipmunks, cottontails, and small rodents (all other small rodents that were not identifiable to a species level), as these species had relatively high naïve occupancy and are likely prey items for many mesocarnivores in Rhode Island. Photo data was processed and organized in the database Camelot (Hendry and Mann 2018). We considered detections to be independent if at least 20 min had elapsed between photos of each species at each site (Burton et al. 2015; Mayer et al. 2022).

**TABLE 1:** Camera trapping effort by survey season and year, in Rhode Island, USA from winter 2018 through winter 2023, with the total number of sites cameras were deployed at, number of cameras deployed, total survey effort (trap nights), average and range of the number of days cameras were active, and the dates cameras were deployed.

Season	Sites	Cameras	Total trap nights	Mean days camera active (range)	Dates
Winter 2018	40	40	3411	85.28 (63–109)	Jan 16–May 18
Summer 2018	100	100	4485	44.85 (13–58)	June 13–Oct 5
Winter 2019	20	50	7128	146.13 (140–151)	Nov 8–Apr 10
Summer 2019	100	200	7802	41.02 (27–52)	June 10–Sep 30
Winter 2020	100	200	9462	49.37 (35–60)	Dec 2–Mar 14
Summer 2020	100	200	8706	45.38 (32–49)	June 8–Sep 18
Winter 2021	200	400	21,536	56.17 (33–71)	Nov 14–Mar 10
Summer 2021	240	480	20,219	44.81 (37–55)	May 26–Sep 10
Winter 2022	239	478	23,277	51.36 (6.5–88.5)	Nov 1–Feb 14
Summer 2022	240	480	20,177	44.49 (5–54)	May 29–Sep 5
Winter 2023	100	200	11,462	59.64 (42–86)	Nov 28–Mar 4

*Note:* From 2019 to 2023, two cameras were deployed per site.

### 2.3 Hypotheses and Predictions

**FIGURE 2:**
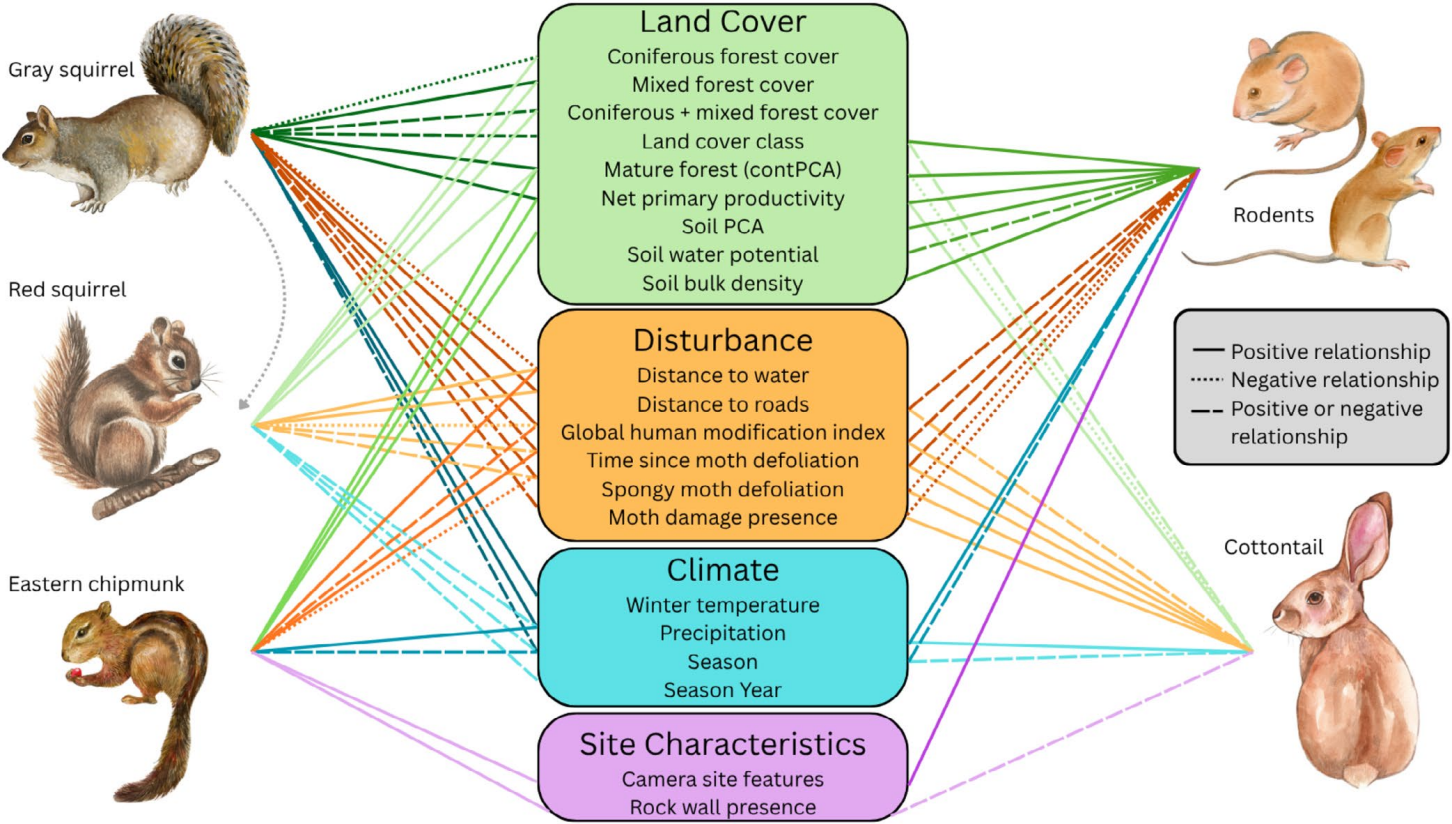
Conceptual figure of predicted responses of gray squirrel, red squirrel, eastern chipmunk, cottontails, and rodents to environmental covariates. Positive relationships are shown with solid lines, negative relationships are shown with dotted lines, and dashed lines indicate the potential for positive or negative relationships. Full descriptions of expected relationships between species' and covariates can be found in Table A1.

We hypothesized that gray squirrel occupancy would be driven by land cover, spongy moth ( *Lymantria dispar* ) damage, primary productivity, and human modification, and red squirrel occupancy would be related to seasonality, precipitation in the previous season, time since defoliation, presence of gray squirrels, land cover, mature contiguous forest, proximity to roads, human modification, and spongy moth damage (Figure 2; Table A1). We expected that cottontail occupancy would be related to seasonality, food availability, spongy moth disturbance, land cover, and human modification. We expected that eastern chipmunk occupancy would be related to precipitation in the previous season, spongy moth damage, land cover, and net primary productivity. For our small rodents, we expected occupancy to be related to food availability, land cover, human modification, soil properties, spongy moth damage, and physical features within camera view. Different small rodents may be active at different times of year (Scott et al. 2022), and food availability varies between seasons, in part due to precipitation. Small rodent results may be best viewed as community‐level responses rather than species‐specific dynamics.

### 2.4 Covariate Selection

We defined 17 different covariates to use in multiple species‐specific models (Table A2). These variables capture land cover composition (e.g., percent coniferous forest, land cover or soil type, etc.), disturbance‐related characteristics (e.g., distance to road, moth damage presence, etc.), climate factors (e.g., precipitation, seasonality), and site‐specific features (e.g., physical feature in front of the camera, gray squirrel presence). As each survey site had two operational cameras, we identified a centroid between the cameras and established two circular buffers of differing radii (50 m, 100 m) to assess the effect of covariate scale on our short‐ranging focal species. Covariates were then extracted either from the central point or were averaged within both buffers to create two scales. We then derived the following variables: land cover class, percent coniferous forest and mixed forest, net primary productivity (NPP), distance to nearest road or freshwater, and a measure of contiguous forest habitat (contPCA; Table A2) to assess components related to land cover. For disturbances, we identified four variables of interest: human modification, moth damage severity, presence of moth damage, and time since defoliation event (TSD). To assess the impact of climate on small mammal occupancy dynamics, we characterized seasonal precipitation patterns in the form of rainfall in summer (June 1–September 30) and snowfall in winter (November 1–March 31). As we believed that physical features would impact small mammal occurrence and detection rates within camera view, we created a feature variable. Reviewing site photos, each site was classified as either having a rock wall, large rocks only (rocky), woody debris, a mix of rocks and woody debris (mixed), or no feature (none). For cottontails, this was reduced to rock wall or no rock wall. Lastly, as we predicted red squirrel occupancy to be impacted by gray squirrel presence, we created a variable representing gray squirrel presence (GSpres) at a site in each season. This variable was derived from our gray squirrel detection histories, where if a gray squirrel was detected at a site during a season, it was assigned a 1; if not, a 0.

### 2.5 Bayesian Occupancy Analysis

For each species, we fit separate dynamic occupancy models in a Bayesian framework (MacKenzie et al. 2003). We fit independent candidate sets of models for each species in the R package “ubms” (Kellner et al. 2022) in R version 4.2.0 (R Core Team 2023). Default diffuse priors were retained from the ‘ubms’ package; for probability parameters (e.g., occupancy, detection, intercepts, regression coefficients) default priors are given as Logistic(0, 1) prior following (Northrup and Gerber 2018; Kellner et al. 2022). To allow direct comparison of coefficients, we mean‐centered and scaled all continuous variables. We fit three parallel chains using random starting values each with 5000 Markov Chain Monte Carlo samples, and a 2500‐sample burn‐in for each model. Parameter convergence was assessed by visually inspecting trace‐plots and using the *R*‐hat statistics. All *R*‐hat values were near 1 (range 1–1.01), indicating convergence (Gelman et al. 2004).

We estimated site‐level initial occupancy (1), colonization (*γ*), extirpation (*ϵ*), and detection (*p*) (MacKenzie et al. 2003). From these values, we derived occurrence at each subsequent primary sampling period (*ψ*_*t*_) that allowed us to calculate the change in occurrence throughout the study from the first summer and winter to the last summer and winter, respectively. Coefficients were considered to have support if the 95% credible interval did not overlap zero. If the difference between the Efficient Leave‐One‐Out Cross‐Validation of models (elpd_diff) was < 4 models had similar predictive performance (Sivula et al. 2025). Models with similar predictive performance were then ranked by model weight, where higher‐weighted models indicated better predictive performance. We did not consider the uncertainty of elpd diff.

## 3 Results

Cameras were deployed for a total of 105,328 trap nights over the study period. Small rodents were detected most often (*n* = 71,421 detections), followed by gray squirrels (*n* = 48,003), red squirrels (*n* = 10,136), eastern chipmunks (*n* = 9983), and cottontails (*n* = 7096). Gray squirrels had the highest consistent naïve occupancy in both summer and winter, while cottontails and eastern chipmunks had the lowest in summer and winter, respectively (Table 2).

**TABLE 2:** Naïve occupancy estimates by species from the first (psi1, psi2) and last (psi7, psi8) summer and winter, respectively of the study.

Species	psi1	psi2	psi7	psi8
Gray squirrel	0.86	0.66	0.95	0.91
Red squirrel	0.45	0.37	0.63	0.31
Cottontail	0.19	0.31	0.26	0.47
Chipmunk	0.26	0.02	0.72	0.02
Rodent	0.28	0.44	0.8	0.69

### 3.1 Gray Squirrels

**FIGURE 3:**
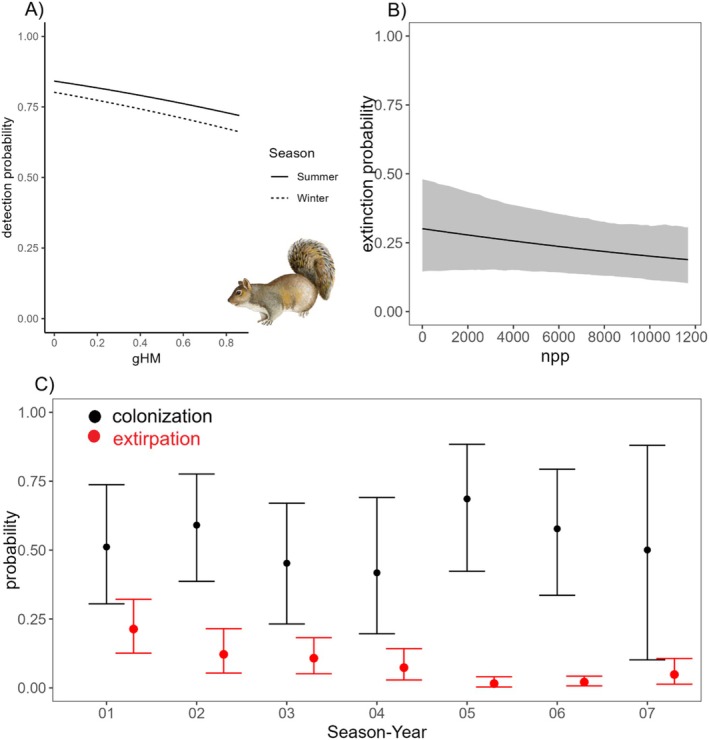
Supported gray squirrel responses based on the top‐ranked model. We show colonization probability based on SeasonYear for comparison, even though it was not supported in the top‐ranked model.

Gray squirrels were the only species with competing alternative models (Models D and E; Table 3) with similar predictive performance (elpd_diff > −4; Table 3). Of the two competing models, model D with the 50 m buffer had the highest cumulative LOO weight (weight = 0.335), and therefore was the most parsimonious and ranked as our top model. Gray squirrels were the only species where a model using the 50 m buffer was the top‐ranked model. In our top‐ranked model, gray squirrel occupancy dynamics were primarily driven by food (in the form of net primary productivity) and annual variation (Table 4, Figure 3c). Extirpation probabilities decreased with increasing net primary productivity ($\beta_{\mathrm{NPP}}$ = −0.30), and were lower from the fourth season onward than in the first year of the study ($\beta_{\text{seasonyr}02}$ through $\beta_{\text{seasonyr}07}$ = −1.38, −3.05, −2.68, −1.74 respectively). Gray squirrel detection probabilities decreased both in winter and as human modification increased ($\beta_{\text{season}\left(\text{winter}\right)}$ = −0.27, $\beta_{\mathrm{gHM}}$ = −0.12; Figure 3a; Table 4). There were no strong predictors of initial occupancy or colonization for gray squirrels in our top‐ranked model (Table 4).

**TABLE 3:** Gray squirrel model ranking.

Model	Occupancy	Detection	Colonization	Extirpation	elpd_diff	weight	rank
A	majority_moth + contPCA + pct.conifer	season + feature + dist_road	season + %mixed	precip*npp + season + %mixed	−27.562	0	8
					−30.445	0	10
B	moth_pres + gHM + pct.conifer	season + feature + dist_road	season + %mixed	precip*npp + season + %mixed	−25.198	0.038	7
					−28.756	0	9
C	gHM + pct.conifer + contPCA	season + feature + dist_road	season + TSD*majority_moth	season + TSD*majority_moth + precip*npp	−7.356	0.269	4
					−8.179	0.005	6
D	gHM + majority_moth + contPCA	season + gHM	precip*npp + seaonyr	precip*npp + seaonyr	−3.175	0.003	3
					−3.498	0.335	1
E	gHM+ contPCA	season + gHM + contPCA	seasonyr	seasonyr	0	0.235	2
					−7.39	0	5
Baseline	~	~	season	season	−35.533	0.114	11

*Note:* Elpd_diff = predictive performance, weight = model fit. Any models with elpd_diff within 4 have similar predictive performance. Models are ranked by elpd_diff and then weight. For each model, the top numbers indicate models using data summarized in 100 m buffers around camera sites, and bottom numbers indicate models using data summarized in 50 m buffers around camera sites.

**TABLE 4:** Beta estimates and standard deviation (SD) for gray squirrel top model, by submodel.

Occupancy (psi)	Beta	SD	LCI	UCI	Supported
intercept	1.40	0.17	1.09	1.74	**
gHM	0.18	0.17	−0.15	0.52	
maj_moth	0.30	0.19	−0.06	0.70	
contPCA	−0.04	0.11	−0.25	0.18	
Detection (*p*)					
intercept	1.29	0.04	1.21	1.37	**
season(winter)	−0.27	0.06	−0.38	−0.15	**
gHM	−0.12	0.03	−0.18	−0.06	**
Colonization (gamma)					
intercept	−0.06	0.44	−0.92	0.82	
precip	−0.65	0.42	−1.49	0.14	
avg_npp	0.30	0.18	−0.03	0.67	
precip:npp	0.28	0.19	−0.07	0.67	
seasonyr02	0.37	0.65	−0.91	1.66	
seasonyr03	−0.19	0.65	−1.49	1.09	
seasonyr04	−0.39	0.81	−2.00	1.18	
seasonyr05	0.90	0.67	−0.37	2.24	
seasonyr06	0.37	0.62	−0.85	1.61	
seasonyr07	0.02	1.09	−2.19	2.14	
Extirpation (epsilon)					
intercept	−1.24	0.29	−1.80	−0.70	**
precip	0.20	0.25	−0.27	0.70	
avg_npp	−0.30	0.11	−0.52	−0.07	**
precip:npp	−0.12	0.14	−0.39	0.14	
seasonyr02	−0.76	0.55	−1.85	0.29	
seasonyr03	−0.89	0.51	−1.88	0.09	
seasonyr04	−1.38	0.63	−2.62	−0.14	**
seasonyr05	−3.05	0.60	−4.33	−1.93	**
seasonyr06	−2.68	0.55	−3.83	−1.64	**
seasonyr07	−1.74	0.50	−2.77	−0.81	**

*Note:* If the 95% confidence interval (LCI, lower confidence interval; UCI, upper confidence interval) does not overlap zero, then the beta estimate is considered supported; indicated by ** in the Supported column.

**FIGURE 4:**
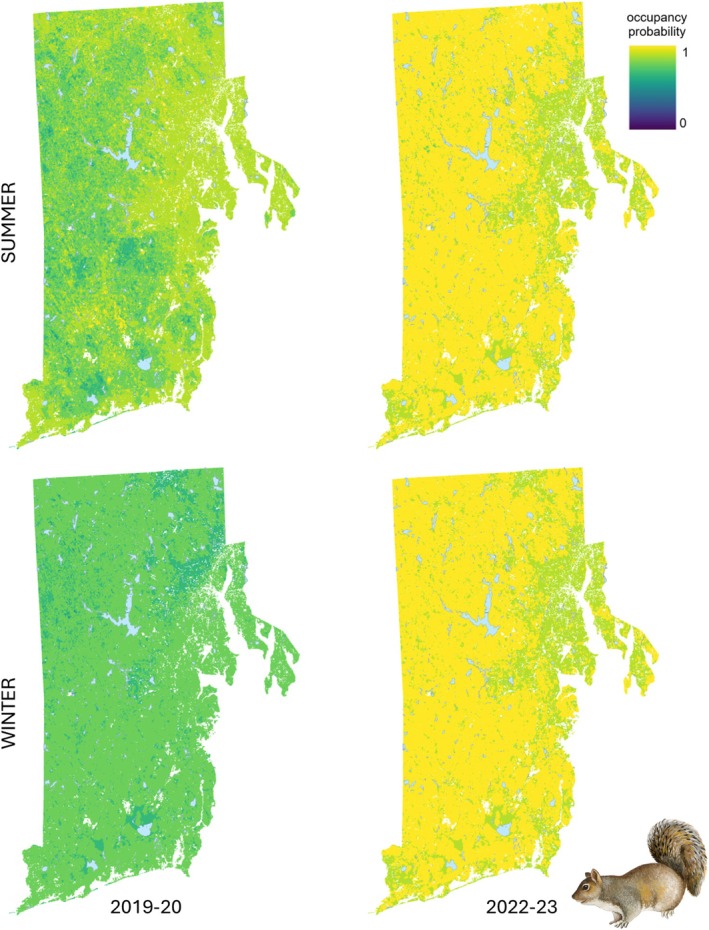
Map of predicted gray squirrel occupancy across Rhode Island. Gray areas indicate sites where magnitude of variables in top model were beyond the limits of our sampled sites, thus no predictions were made to those grid cells.

Gray squirrel site‐specific occupancy increased by 28.3% from the first to last year of the study, with the highest change in occupancy between the first and last winter (36.2%; Table 5). Using our model to predict statewide occupancy trends, gray squirrel occupancy increased by an average of 20.5% from 2019 to 2023, with winter season again having the largest change in occupancy (25.7%; Table 5). In the last year of the study, gray squirrels appeared to have uniformly high occupancy throughout Rhode Island (Figure 4).

**TABLE 5:** Change in site‐specific occupancy for each species, from 2019 to 2023, in each season.

Species	Summer	Winter	Overall	Summer change	Winter change	Overall change
Gray squirrel	1.20	1.36	1.28	20% increase	36% increase	28% increase
Red squirrel	1.15	1.09	1.12	15% increase	9% increase	12% increase
Cottontail	1.35	1.09	1.22	35% increase	9% increase	22% increase
Chipmunk	2.56	2.92	2.74	156% increase	192% increase	174% increase
Rodent	1.31	1.60	1.46	31% increase	60% increase	46% increase

*Note:* A lambda value of 1 indicates no change; negative values = decline, positive values = increase.

### 3.2 Red Squirrel

**FIGURE 5:**
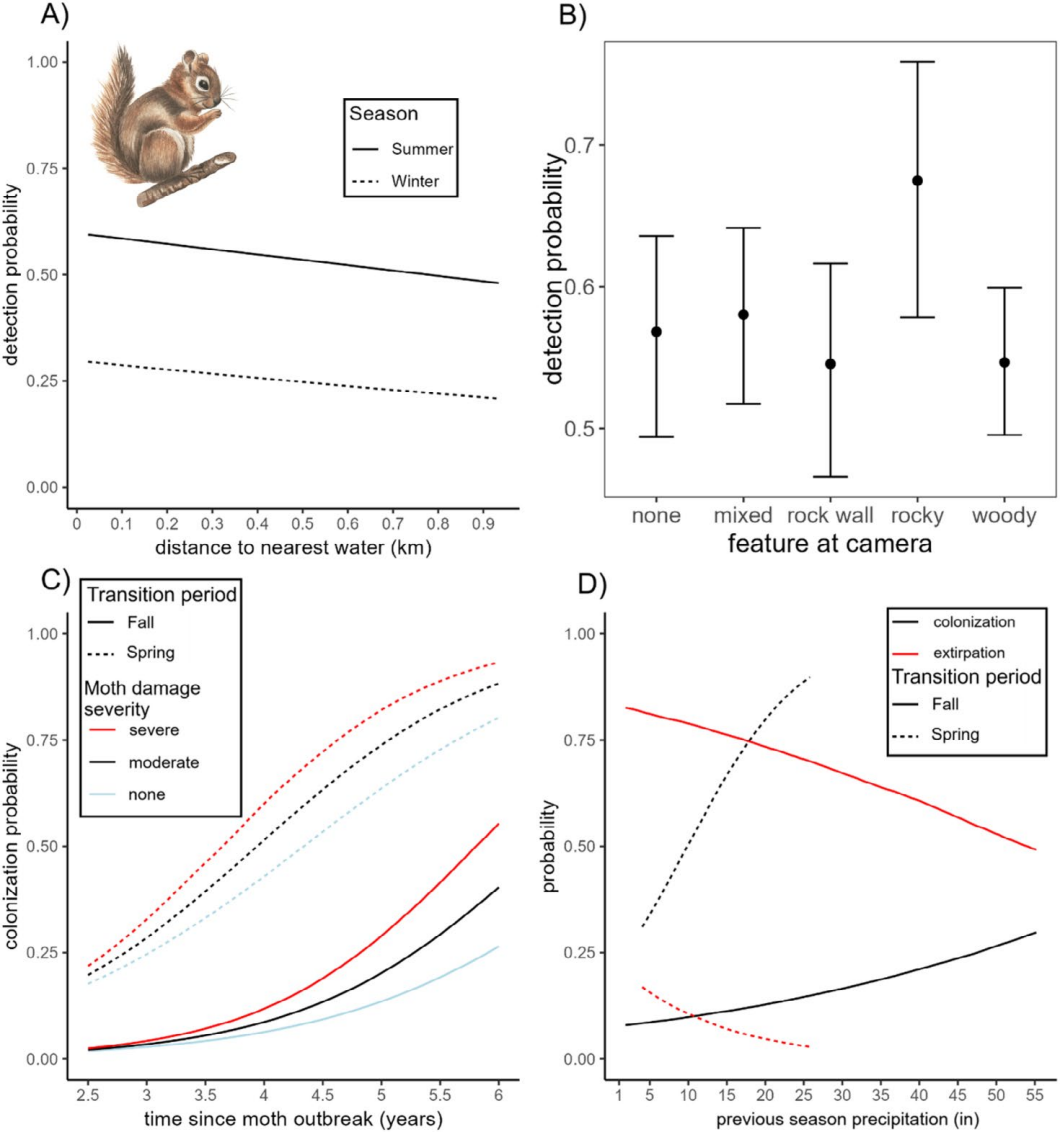
Supported red squirrel responses based on the top‐ranked model.

Red squirrels had two competing models with similar predictive performance, Model B with the 50 m and the 100 m buffer. Model B with a 100 m buffer had the highest cumulative LOO weight (weight = 0.418) and was therefore ranked as our top model for red squirrel (Table 6). Red squirrel occupancy dynamics were primarily driven by the percentage of coniferous forest and the amount of human modification at a site (Table 7). Colonization probability was higher in the transition period from winter to summer (i.e., spring; $\beta_{\text{spring transition}}$= 1.25) and sites were more likely to be colonized with increasing percentage of coniferous forest ($\beta_{\text{gHMpct}.\text{conifer}}$ = 0.23). Red squirrels were more likely to be extirpated from sites with increasing levels of human modification ($\beta_{\mathrm{gHM}}$ = −0.36), particularly during the transition from summer to winter (i.e., autumn; $\beta_{\text{spring transition}}$ = −1.68) (Figure 5; Table 7). Detection probabilities were driven by seasonal variation and the presence of gray squirrels at a site (Figure 5). Gray squirrel presence had a negative impact on red squirrel detection probability ($\beta_{\mathrm{gsq}\left(\text{present}\right)}$ = −0.24) (Figure 5; Table 7). Detection probabilities were higher from the fifth season through the eighth season compared to the first survey season ($\beta_{\text{seasonyr}02}$ through $\beta_{\text{seasonyr}08}$ = 0.54, 0.92, 0.88, 0.59, respectively). There were no strong predictors of initial site occupancy in our top‐ranked model (Table 7).

**TABLE 6:** Red squirrel model ranking.

Model	Occupancy	Detection	Colonization	Extirpation	elpd_diff	weight	rank
A	pct.conifer + gsqPA + dist_water	seasonyr + gsquirrel	season + majority_moth*pct.conifer + precip + contPCA	season + majority_moth*pct.conifer + precip + contPCA	−6.982	0	5
					−7.746	0	6
B	pct.conifer + gsqPA + dist_water + gHM	seasonyr + gsquirrel	season + majority_moth*pct.conifer + precip + gHM	season + majority_moth*pct.conifer + precip + gHM	0	0.418	1
					−1.317	0	2
C	contPCA + dist_road + gsqPA + pct.conifer	gsquirrel + Season	gHM *season + contPCA	gHM *season + contPCA	−39.1553	0.001	9
					−38.166	0.116	8
D	pct.conifer + gsqPA	gsquirrel + SeasonYr	Season + Precip + TSD*majority_moth	Season + Precip + TSD*majority_moth	−5.461	0.142	4
					−5.374	0.058	3
E	pct.conifer + gsqPA	gsquirrel + season	season*precip	season*precip	−40.564	0	11
					−40.402	0	10
Baseline	~	~	Season	Season	−32.133	0.265	7

*Note:* Elpd_diff = predictive performance, weight = model fit. Any models with elpd_diff within 4 have similar predictive performance. Models are ranked by elpd_diff and then weight. For each model, the top numbers indicate models using data summarized in 100 m buffers around camera sites, and bottom numbers indicate models using data summarized in 50 m buffers around camera sites.

**TABLE 7:** Beta estimates and standard deviation (SD) for red squirrel top model, by submodel.

Occupancy (psi)	Beta	SD	LCI	UCI	Supported
intercept	−0.03	0.17	−0.35	0.3	
pct.conifer	−0.03	0.15	−0.32	0.26	
gsq(present)	0.36	0.32	−0.25	1	**
dist_water	0.2	0.16	−0.11	0.53	
gHM	0.28	0.15	−0.02	0.59	
Detection (*p*)					
intercept	−0.18	0.16	−0.5	0.14	
seasonyr02	−0.28	0.21	−0.69	0.12	
seasonyr03	0.27	0.19	−0.1	0.65	
seasonyr04	0.31	0.17	−0.02	0.64	
seasonyr05	0.54	0.16	0.23	0.84	**
seasonyr06	0.92	0.16	0.6	1.24	**
seasonyr07	0.88	0.16	0.58	1.2	**
seasonyr08	0.59	0.21	0.18	1.01	**
gsq(present)	−0.24	0.09	−0.42	−0.06	**
Colonization (gamma)					
intercept	−1.74	0.24	−2.24	−1.29	**
transition(spring)	1.25	0.32	0.64	1.92	**
maj_moth	0.01	0.12	−0.23	0.23	
pct_conifer	0.23	0.12	0	0.47	*
maj_moth*pct_conifer	0.08	0.16	−0.24	0.4	
precip	−0.16	0.16	−0.46	0.15	
gHM	0.21	0.13	−0.05	0.47	
Extirpation (epsilon)					
intercept	−0.33	0.17	−0.66	0.01	**
transition(spring)	−1.68	0.32	−2.32	−1.08	**
maj_moth	−0.1	0.13	−0.35	0.14	
pct_conifer	−0.08	0.12	−0.32	0.16	
maj_moth*pct_conifer	0.24	0.15	−0.05	0.54	
precip	−0.05	0.14	−0.32	0.21	
gHM	−0.36	0.11	−0.58	−0.15	**

*Note:* If the 95% confidence interval (LCI = lower confidence interval, UCI = upper confidence interval) does not overlap zero, then the beta estimate is considered supported; indicated by ** in the Supported column.

**FIGURE 6:**
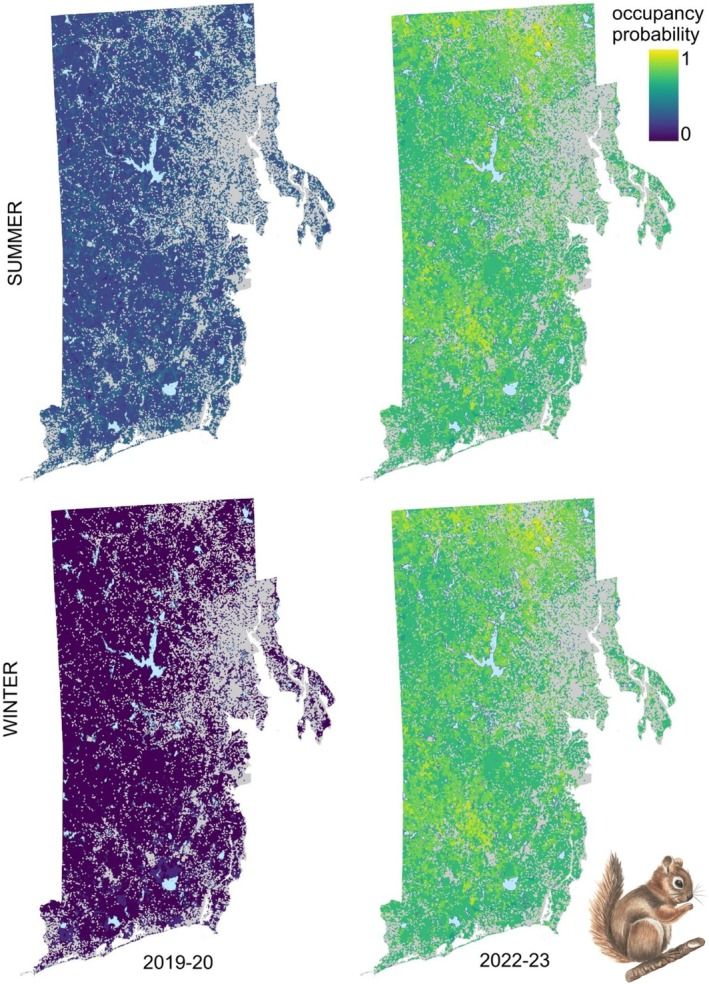
Map of predicted red squirrel occupancy across Rhode Island. Gray areas indicate sites where magnitude of variables in top model were beyond the limits of our sampled sites, thus no predictions were made to those grid cells.

Red squirrel site‐specific occupancy remained stable throughout the study with an average increase of 11.7% from the first to last year (14.7% increase across summers, 8.6% increase across winters) (Table 6). Using the top model to predict statewide occupancy, red squirrel occupancy remained stable with only a minimal decline (0.2%; Table 5) from the first to last surveys. Predicted statewide occupancy probabilities for red squirrels across the survey seasons were higher in the summer season than the winter season but remained largely stable from the summer 2019 to 2022 and winter 2019 to 2023 (Figure 6).

### 3.3 Cottontails

**FIGURE 7:**
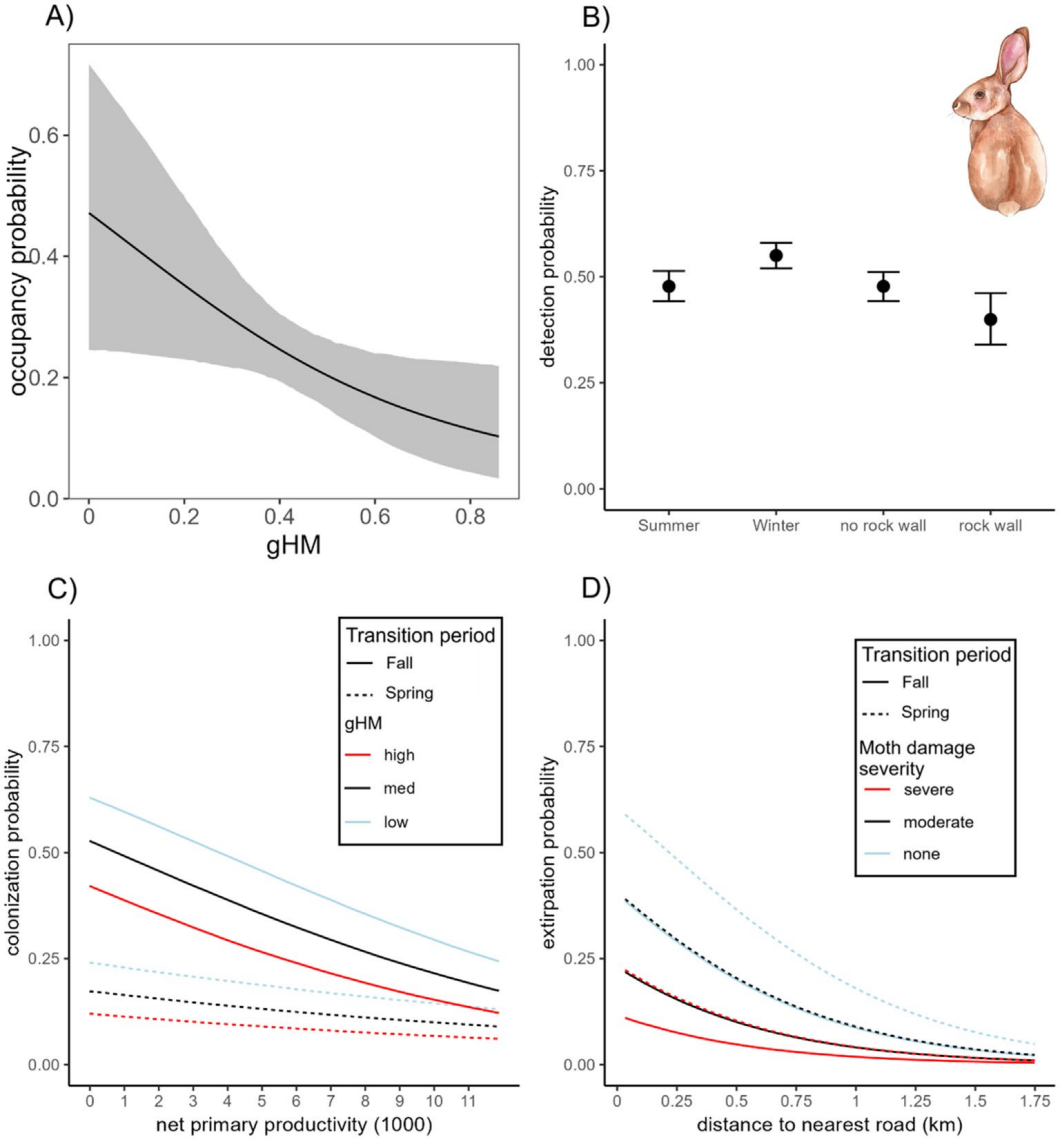
Supported cottontail responses based on the top‐ranked model.

The top model for cottontails, Model F with a 100 m buffer (Table 8), had the best predictive performance and the second‐best fit (weight = 0.225). In our top‐ranked model for cottontails, initial occupancy was best predicted by the global human land modification index ($\beta_{\mathrm{gHM}}$ = −0.37; Table 9). Cottontail occupancy probability decreased as human land modification increased (Figure 7a). Cottontail detection probability was higher in the winter months ($\beta_{\text{season}\left(\text{winter}\right)}$ = −0.29; Table 9), but decreased with the presence of rock walls within the camera frame ($\beta_{\text{rock wall}}$ = −0.29; Table 9; Figure 7b). Cottontail colonization probability decreased during the spring transition from winter to summer ($\beta_{\text{spring transition}}$ = −1.07; Table 9), and at higher levels of human modification ($\beta_{\mathrm{gHM}}$ = −0.21; Table 9) and net primary productivity ($\beta_{\mathrm{NPP}}$ = −0.21; Table 9; Figure 7c). Additionally, extirpation probabilities were higher in the spring transition period ($\beta_{\text{spring transition}}$ = 0.84; Table 9) than in the autumn, and decreased at sites farther away from roads ($\beta_{\text{distance to roads}}$ = −0.40; Table 9; Figure 7d), and as the severity of moth damage increased ($\beta_{\mathrm{maj}\_\text{moth}}$ = −0.41; Table 9; Figure 7d).

**TABLE 8:** Cottontail model ranking.

Model	Occupancy	Detection	Colonization	Extirpation	elpd_diff	weight	rank
A	dist_roads + contPCA	Season + dist_roads	TSD*majority_moth + precip*npp + Season	TSD*majority_moth + precip*npp + Season	−8.49	0.145	9
					−14.182	0	15
B	majority_moth + dist_road + contPCA	Season + dist_roads	precip*npp + Season	precip*npp + Season	−11.765	0	17
					−16.226	0	18
C	moth_pres + dist_road + contPCA	Season + dist_roads	precip*npp + Season	precip*npp + Season	−11.472	0	16
					−16.226	0	19
D	moth_pres + dist_road + contPCA	Season + dist_roads + rock_wall	dist_roads + gHM + Season	dist_roads + gHM + Season	−10.588	0.001	13
					−10.133	0	11
E	moth_pres + dist_road + contPCA	Season + dist_roads + rock_wall	dist_roads + gHM + Season + contPCA	dist_roads + gHM + Season + contPCA	−10.619	0.096	14
					−10.133	0	12
F	lc_class + gHM	Season + rock_wall	Season + maj_moth + npp*Precip + gHM	Season + maj_moth + dist_roads	0	0.225	1
					−4.824	0.056	2
G	gHM	Season + rock_wall	Season + gHM + contPCA	Season + gHM + contPCA	−7.73	0	4
					−6.757	0.306	3
Baseline	~	~	Season	Season	−9.488	0.171	10

*Note:* Elpd_diff = predictive performance, weight = model fit. Any models with elpd_diff within 4 have similar predictive performance. Models are ranked by elpd_diff and then weight. For each model, the top numbers indicate models using data summarized in 100 m buffers around camera sites, and bottom numbers indicate models using data summarized in 50 m buffers around camera sites.

**TABLE 9:** Beta estimates and standard deviation (SD) for cottontail top model, by submodel.

Occupancy (psi)	Beta	SD	LCI	UCI	Supported
intercept	−1.26	0.17	−1.59	−0.94	**
lc(wetland)	−0.6	1.54	−4.01	2.13	
lc(young forest)	0.46	0.73	−1.02	1.83	
gHM	−0.37	0.17	−0.71	−0.04	**
Detection (*p*)					
intercept	−0.09	0.07	−0.23	0.05	
season(winter)	0.29	0.09	0.12	0.47	**
rock wall	−0.33	0.13	−0.59	−0.06	**
Colonization (gamma)					
intercept	−1.07	0.16	−1.39	−0.75	**
transition(spring)	−1.07	0.3	−1.68	−0.5	**
npp	−0.21	0.1	−0.4	−0.01	**
precip	−0.04	0.14	−0.3	0.23	
npp*precip	−0.12	0.12	−0.36	0.1	
gHM	−0.21	0.11	−0.42	−0.01	*
maj_moth	0.17	0.1	−0.04	0.34	
Extirpation (epsilon)					
intercept	−1.15	0.21	−1.57	−0.76	**
transition(spring)	0.84	0.26	0.33	1.34	**
dist_roads	−0.4	0.18	−0.78	−0.07	**
maj_moth	−0.41	0.15	−0.73	−0.12	**

*Note:* If the 95% confidence interval (LCI, lower confidence interval; UCI, upper confidence interval) does not overlap zero, then the beta estimate is considered supported; indicated by ** in the Supported column.

**FIGURE 8:**
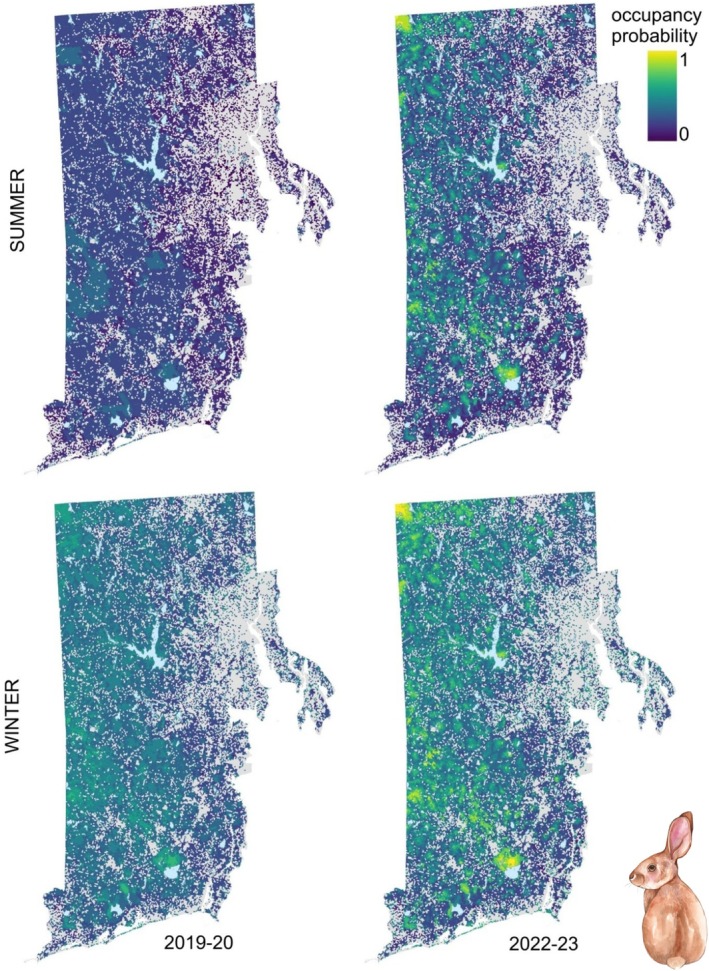
Map of predicted cottontail occupancy across Rhode Island. Gray areas indicate sites where magnitude of variables in top model were beyond the limits of our sampled sites, thus no predictions were made to those grid cells.

Cottontail site‐specific occupancy increased by 22.0% from the first year of the study to the last (Table 5). The highest change in occupancy was a 34.8% increase from the first to the last summer of the study period (Table 5). Predicted statewide occupancy increased by 23.8% from 2019 to 2023, with the largest seasonal increase in summer of 38.5% (Table 5; Figure 8). Our statewide predictions show increases primarily in the western portions of the state away from the coastline (Figure 8).

### 3.4 Eastern Chipmunk

**FIGURE 9:**
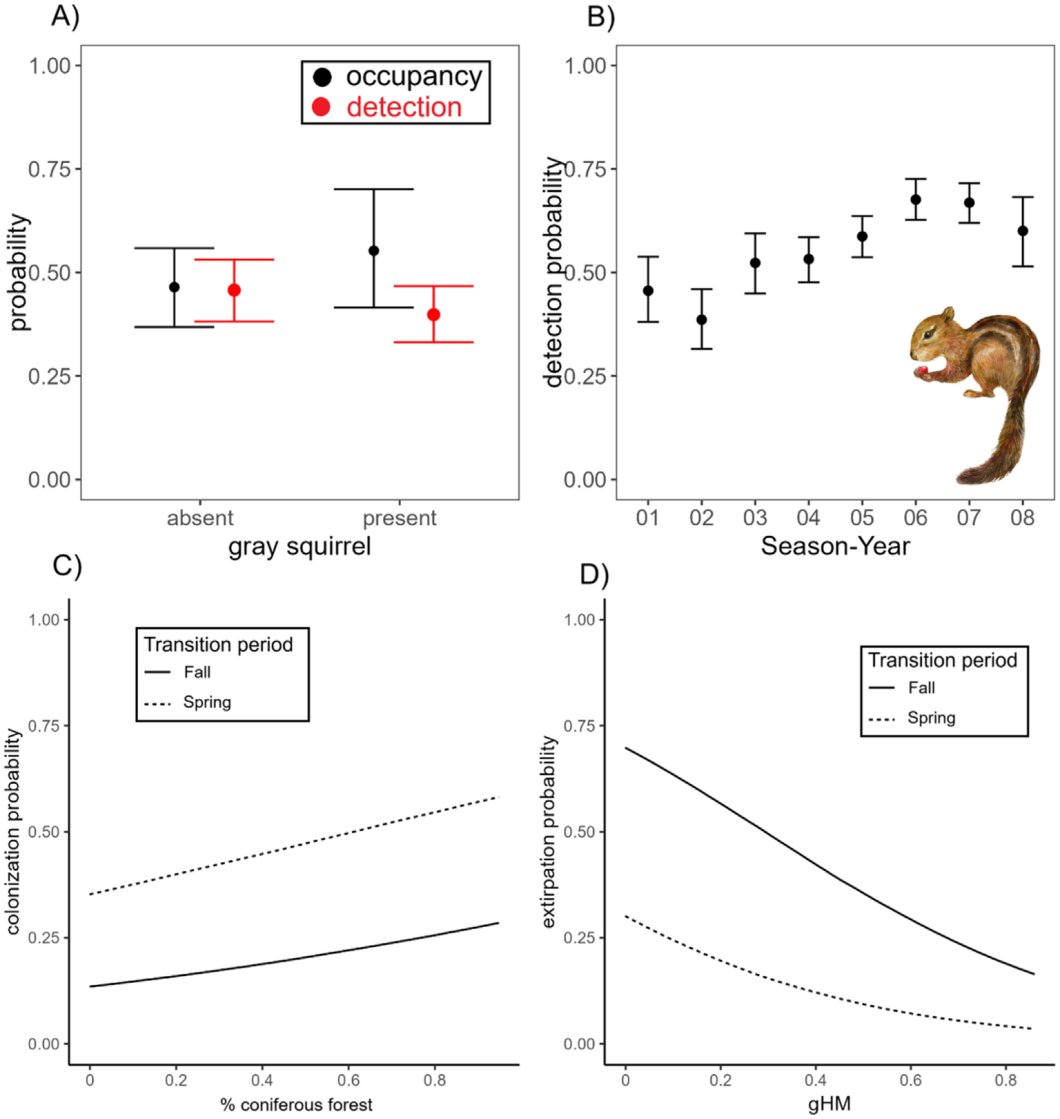
Supported eastern chipmunk responses based on the top‐ranked model.

Eastern chipmunks had a single top‐ranked model (Model A; Table 10), with no competing alternatives (elpd_diff > 4 for all other models). Our top model with the 100 m buffer had the highest cumulative LOO weight (weight = 0.425) and was also considered the best‐fitting model. The 50 m version of the same model ranked second (weight = 0.190) but received less support (Table 10). In our top‐ranked model, eastern chipmunk occupancy dynamics were primarily influenced by seasonal variation, time since defoliation, moth damage severity, and precipitation (Table 11; Figure 9). There were no strong predictors of initial occupancy (Table 11). Detection probabilities decreased in winter ($\beta_{\text{season}\left(\text{winter}\right)}$ = −1.18) and increased at sites with rocky features ($\beta_{\text{features}\left(\text{rocky}\right)}$ = 0.45). Colonization probabilities increased with longer time since defoliation ($\beta_{\mathrm{TSD}}$ = 1.26), and during the spring transition period ($\beta_{\text{spring transition}}$= 1.76) (Table 11; Figure 9c), but declined with higher precipitation ($\beta_{\text{precipitation}}$= −0.47; Figure 9d). Extirpation probabilities were lower in spring compared to the autumn transition ($\beta_{\text{spring transition}}$ = −3.18).

**TABLE 10:** Eastern chipmunk model ranking.

Model	Occupancy	Detection	Colonization	Extirpation	elpd_diff	weight	rank
A	features + soiPCA + contPCA	season + precip + features + dist_water	precip*npp + TSD*majority_moth + dist_water + season	precip*npp + TSD*majority_moth + dist_water + season	0	0.425	1
					−1.855	0.19	2
B	features + soiPCA + contPCA + gHM	Season + gHM + features + dist_water	precip + dist_water + season	precip + dist_water + season	−18.197	0	6
					−17.969	0.164	5
C	rock_wall + soilPCA	Season + rock_wall + dist_water	precip*npp + season	precip*npp + season	−13.744	0.017	4
					−13.476	0	3
Baseline	~	~	Season	Season	−54.895	0.203	7

*Note:* Elpd_diff = predictive performance, weight = model fit. Any models with elpd_diff within 4 have similar predictive performance. Models are ranked by elpd_diff and then weight. For each model, the top numbers indicate models using data summarized in 100 m buffers around camera sites, and bottom numbers indicate models using data summarized in 50 m buffers around camera sites.

**TABLE 11:** Beta estimates and standard deviation (SD) for eastern chipmunk top model, by submodel.

Occupancy (psi)	Beta	SD	LCI	UCI	Supported
intercept	−0.89	0.4	−1.69	−0.12	**
features(mixed)	−0.15	0.53	−1.2	0.88	
features(rock wall)	0.01	0.57	−1.11	1.12	
features(rocky)	−0.44	0.8	−2.14	1.05	
features(woody)	−0.11	0.45	−1	0.8	
contPCA	−0.11	0.12	−0.34	0.11	
soilPCA	0	0.12	−0.23	0.22	
Detection (*p*)					
intercept	0.26	0.14	0	0.54	*
season(winter)	−1.18	0.17	−1.51	−0.85	**
precip	0.06	0.06	−0.07	0.18	
dist_water	−0.09	0.05	−0.18	0	*
features(mixed)	0.04	0.16	−0.26	0.34	
features(rock wall)	−0.1	0.17	−0.44	0.23	
features(rocky)	0.45	0.24	0	0.92	**
features(woody)	−0.1	0.13	−0.36	0.16	
Colonization (gamma)					
intercept	−1.87	0.27	−2.4	−1.35	**
maj_moth	0.19	0.1	−0.01	0.39	*
TSD	1.26	0.19	0.89	1.65	**
maj_moth*TSD	0.09	0.17	−0.24	0.43	
precip	−0.47	0.18	−0.82	−0.13	**
npp	−0.04	0.11	−0.26	0.17	
precip*npp	−0.15	0.11	−0.38	0.07	
dist_water	−0.09	0.09	−0.27	0.09	
transition(spring)	1.76	0.35	1.06	2.45	**
Extirpation (epsilon)					
intercept	1.21	0.31	0.62	1.82	**
maj_moth	−0.12	0.19	−0.49	0.26	
TSD	0.02	0.26	−0.49	0.53	
maj_moth*TSD	−0.22	0.29	−0.79	0.34	
precip	0.13	0.26	−0.37	0.64	
npp	0.13	0.22	−0.26	0.61	
precip*npp	−0.12	0.26	−0.68	0.34	
dist_water	−0.06	0.17	−0.4	0.27	
transition(spring)	−3.18	0.57	−4.36	−2.12	**

*Note:* If the 95% confidence interval (LCI, lower confidence interval; UCI, upper confidence interval) does not overlap zero, then the beta estimate is considered supported; indicated by ** in the Supported column.

**FIGURE 10:**
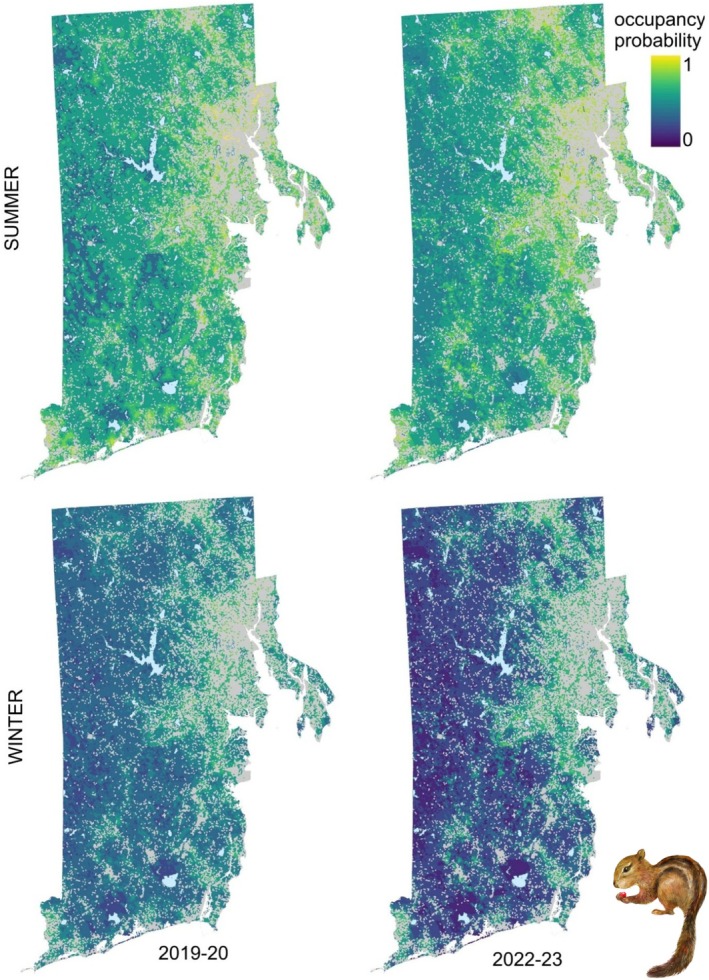
Map of predicted eastern chipmunk occupancy across Rhode Island. Gray areas indicate sites where magnitude of variables in top model were beyond the limits of our sampled sites, thus no predictions were made to those grid cells.

Eastern chipmunk site‐specific occupancy increased by 174% from the first to last year of the study, with the highest change in occupancy between the first and last winter (192%; Table 5). Using our model to predict statewide occupancy trends, eastern chipmunk occupancy increased by an average of 173% from 2019 to 2023, with winter again showing the largest change in occupancy (202%; Table 5). In the final year of the study, eastern chipmunks appeared to have high occupancy across Rhode Island (Figure 10).

### 3.5 Small Rodents

**FIGURE 11:**
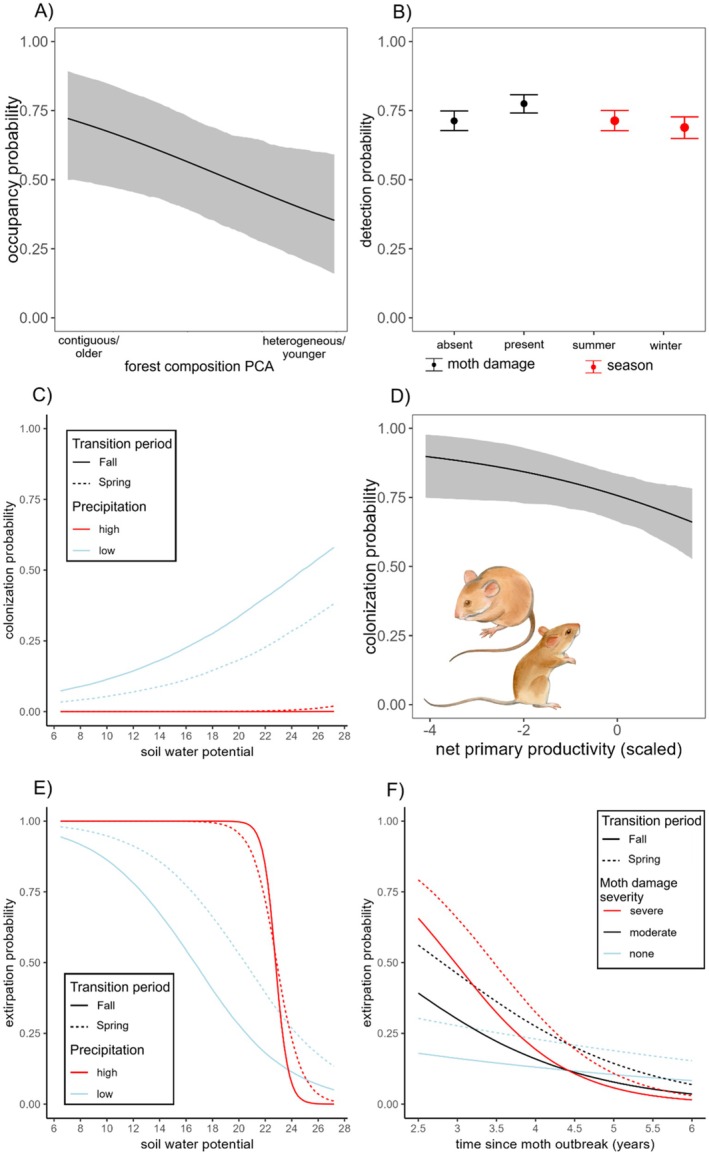
Supported small rodent responses based on the top‐ranked model.

The top model for the small rodent community was a model with the 100 m buffer (weight = 0.237; Model A, Table 12). In this top model, small rodent community initial occupancy was primarily influenced by forest structure (Table 13; Figure 11a), with higher occupancy probabilities in contiguous, older forests ($\beta_{\text{contPCA}}$ = −0.23). Additionally, detection was influenced by season and moth damage, with higher detection rates in moth‐damaged areas ($\beta_{\text{moth presence}}$ = 033) and in summer ($\beta_{\text{season}\left(\text{winter}\right)}$ = −0.22; Figure 11b). Colonization and extinction submodels indicated more complex drivers (Table 13). Apparent colonization was influenced primarily by time since defoliation ($\beta_{\mathrm{TSD}}$ = 0.05), average seasonal precipitation from the previous season ($\beta_{\text{precipitation}}$ = −0.42), mean net primary productivity ($\beta_{\mathrm{NPP}}$= −0.29), and seasonality ($\beta_{\text{spring transition}}$ = −0.28). For small rodents, apparent colonization was highest in areas with lower net primary productivity and lower winter precipitation (Figure 11c,d). Apparent extirpation was primarily driven by seasonality ($\beta_{\text{spring transition}}$ = 1.07), and interactions between time since defoliation and moth damage severity ($\beta_{\text{moth}*\mathrm{TSD}}$ = −0.35) and between average seasonal precipitation from the previous season and mean soil water potential ($\beta_{\text{precip}*\text{water potential}}$ = −0.22). Apparent extirpation probability was highest at low to moderate soil water potential, particularly when there was high precipitation in the previous season (Figure 11e,f).

**TABLE 12:** Small rodent model ranking.

Model	Occupancy	Detection	Colonization	Extirpation	elpd_diff	weight	rank
A	features2 + gHM + contPCA + soilPCA	soilPCA + season + moth_pres + features2	season + precip*watpot + npp + TSD*majority_moth	season + precip*watpot + npp + TSD*majority_moth	0	0.237	1
					−5.145	0.189	3
B	features2 + gHM + dist_roads + contPCA	season + dist_roads + features2	dist_roads + gHM + season + contPCA	dist_roads + gHM + season + contPCA	−33.582	0	7
					−39.328	0.001	9
C	features2 + gHM + dist_roads + contPCA + moth_pres	season + dist_roads + features + moth_pres	dist_roads + TSD*majority_moth + season + gHM + contPCA	dist_roads + TSD*majority_moth + season + gHM + cont_PCA	−4.639	0.168	2
					−9.246	0.001	4
D	features2 + lc_class + bulkden + soilPCA	season + npp + features2	season + precip*watpot	season + precip*watpot	−32.363	0	6
					−35.093	0	8
Baseline	~	~	Season	Season	−21.946	0.404	5

*Note:* Elpd_diff = predictive performance, weight = model fit. Any models with elpd_diff within 4 have similar predictive performance. Models are ranked by elpd_diff and then weight. For each model, the top numbers indicate models using data summarized in 100 m buffers around camera sites, and bottom numbers indicate models using data summarized in 50 m buffers around camera sites.

**TABLE 13:** Beta estimates and standard deviation (SD) for small rodent top model, by submodel.

Occupancy (psi)	Beta	SD	LCI	UCI	Supported
intercept	0.32	0.37	−0.39	1.05	
features2(other)	0.24	0.4	−0.06	1.02	
features2(rock wall)	−0.03	0.52	−1.06	1	
gHM	0.08	0.14	−0.2	0.37	
contPCA	−0.23	0.1	−0.44	−0.03	**
soilPCA	0.07	0.1	−0.12	0.26	
Detection (*p*)					
intercept	0.91	0.09	0.73	1.1	**
season(winter)	−0.12	0.06	−0.23	−0.01	**
soilPCA	−0.01	0.02	−0.04	0.03	
moth_presence	0.33	0.06	0.21	0.45	**
features2(other)	−0.08	0.09	−0.27	0.1	
features2(rock wall)	−0.13	0.13	−0.37	0.12	
Colonization (gamma)					
intercept	1.31	0.23	0.87	1.77	**
maj_moth	0.02	0.16	−0.29	0.33	
TSD	1.19	0.2	0.81	1.58	**
moth*TSD	0.05	0.2	−0.32	0.44	
precip	−0.42	0.18	−0.78	−0.06	**
watpot	0.16	0.15	−0.13	0.45	
precip*watpot	0.06	0.14	−0.21	0.35	
npp	−0.29	0.14	−0.58	−0.02	**
transition(spring)	−0.82	0.35	−1.49	−0.14	**
Extirpation (epsilon)					
intercept	−2.11	0.23	−2.57	−1.69	**
maj_moth	0.05	0.11	−0.17	0.26	
TSD	−0.58	0.19	−0.94	−0.21	**
moth*TSD	−0.35	0.18	−0.71	0.01	*
precip	0.27	0.18	−0.08	0.61	
watpot	0.1	0.1	−0.09	0.29	
precip*watpot	−0.22	0.1	−0.42	−0.02	**
npp	0.09	0.1	−0.11	0.29	
transition(spring)	1.07	0.34	0.42	1.73	**

*Note:* If the 95% confidence interval (LCI, lower confidence interval; UCI, upper confidence interval) does not overlap zero, then the beta estimate is considered supported; indicated by ** in the Supported column.

**FIGURE 12:**
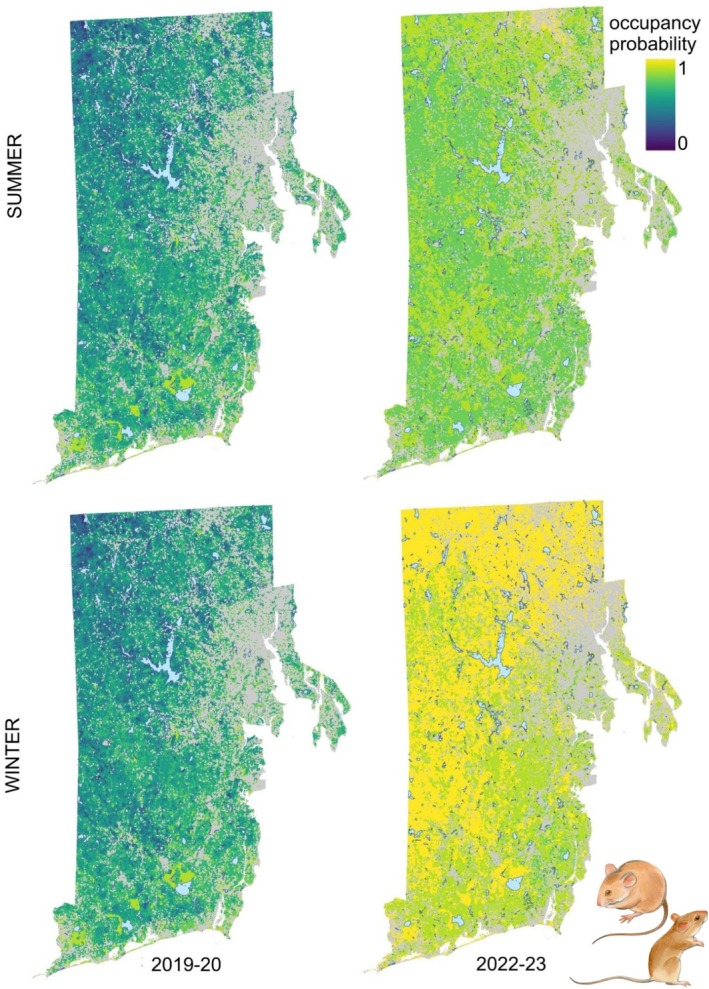
Map of predicted small rodent occupancy across Rhode Island. Gray areas indicate sites where magnitude of variables in top model were beyond the limits of our sampled sites, thus no predictions were made to those grid cells.

From 2019 to 2023, small rodent community occupancy increased across Rhode Island. In that period, our estimated site‐specific small rodent community occupancy increased by 45.9% and state‐wide occupancy increased by 53.1% (Table 5). Increases in winter primarily drove the magnitude of average study‐length changes in occupancy. Small rodent site‐specific winter occupancy increased 60.4% and state‐wide occupancy increased 63.2%. Despite trends of increasing state‐wide occupancy, small rodent occupancy did not increase uniformly (Figure 12). For example, several parts of the state (e.g., the northwestern corner of Rhode Island) shifted from occupancy probabilities near zero to approaching one across summers. In winter, the small rodent community shifted from relatively dispersed across the state, with patches of higher occupancy in the south relative to the north, to higher occupancy away from the coastline (the southern and eastern parts of Rhode Island; Figure 12).

## 4 Discussion

The increased occupancy across all species from 2019 to 2023, except for red squirrels, which showed a minor decline but remained essentially stable, aligned with our hypotheses that these species are relatively generalist in nature, responding to a variety of conditions including food availability, human modification, and forest structure, as well as having seasonal variation in populations. Over the study period, mesocarnivore occupancy probabilities declined in Rhode Island (Ganoe et al. 2024); however, we did not see a similar decline in occupancy probabilities of potential prey species. This could suggest that prey declines are not the primary cause of mesocarnivore declines in Rhode Island; however, further study relating changes in prey and mesocarnivore dynamics are warranted. In other areas of New England, for example, the relationship between occupancy dynamics of cottontails and potential predators varied spatially and by predator species (Testerman and Hapeman 2022).

Common, widespread species can drive ecosystem delivery and biodiversity patterns but (Gaston 2010; Winfree et al. 2015) may be overlooked or studied less than species which are equally widespread but threatened or endangered (Readyhough et al. 2025; Virtanen and Moilanen 2023). There are many historical cases of common species being driven towards extinction because they were overlooked or overexploited (Gaston 2010; Gaston and Fuller 2008), and for common species a proportionally small decline equates to a large loss of individuals, biomass, and species interactions (Baker et al. 2019). Conversely, some native species can become hyperabundant “native invaders,” having implications for conservation (Brown et al. 2025), human health (Bastard et al. 2024; Shultz et al. 2023; Wallace et al. 2014), or human‐wildlife conflict or interactions (Fidino et al. 2022; Lazure and Weladji 2024).

Our study species' are ubiquitous on college campuses (Peplinski and Brown 2020), urban parks, and gardens (Tranquillo et al. 2024), are widely hunted (Steiner and Huettmann 2023); are perceived positively and negatively in terms of their interactions with humans (Benson 2013; Hohbein and Mengak 2018; Holm 2012); and can serve as vectors for zoonotic diseases and parasites with implications for human health (Bisanzio et al. 2015; Diuk‐Wasser et al. 2026; Schiff et al. 2025; Weyna et al. 2024). In Rhode Island, and New England more broadly, eastern cottontails are an introduced species which compete with and lower the abundance of the native imperiled New England cottontail ( *Sylvilagus transitionalis* ; Bischoff et al. 2023). In the northern hemisphere, small rodents and their cyclical population dynamics play a key role in linking vegetation dynamics and predators in food webs, and also have implications for humans such as crop damage, disease spread, or effects on game species that rely on rodents as a prey source (Boonstra and Krebs 2012; Soininen and Neby 2024; Wood and Lafferty 2013).

These insights, alongside the impact of changing New England forests on mammal communities and the impacts of species with boom‐and‐bust population cycles, indicate the utility of assessing species bycatch from camera arrays designed initially for one or a few target species. Long‐term camera trapping efforts that include the most heavily modified areas may be necessary to fully understand occupancy dynamics of these species in Rhode Island.

### 4.1 Gray Squirrels

Our findings support the idea that gray squirrels are generalists (Amspacher et al. 2019), and can easily adapt to areas with mass tree mortality. As such, we did not find any evidence of forest type as an indicator of gray squirrel occurrence, despite that elsewhere, gray squirrel densities were lower in coniferous forests (Bertolino et al. 2008) and higher in mixed or deciduous forests (Bertolino et al. 2008; Fischer and Holler 1991; Riege 1991). We may not have found an effect of forest type on gray squirrel occupancy because coniferous forest covers only a small fraction of the total land area of Rhode Island and coniferous forest patches were often small compared to deciduous forest patches. Similarly, we found no support that spongy moth damage impacted gray squirrels. While masting tree mortality would be expected to produce less forage for gray squirrels in the form of nuts (e.g., acorns, walnuts), forest regeneration occurring after spongy moth damage may provide alternative food sources. High net primary productivity areas (e.g., early successional forest) were important in gray squirrel site fidelity, as gray squirrels were less likely to leave higher productivity sites.

Gray squirrels can thrive in human‐dominated urban and suburban landscapes (Anthonysamy et al. 2025; Tranquillo et al. 2024), often at high densities, thus making them potentially easier to detect on cameras (Engel et al. 2020; Gonzales 2005; Tranquillo et al. 2024). However, we did not find a strong relationship between gray squirrel occupancy and human modification. Instead, we found that gray squirrels were slightly more difficult to detect with increasing human modification. We may not have found a strong relationship between gray squirrel occupancy and human modification due to our sampling design, which intentionally avoided placing cameras in the most highly developed areas of Rhode Island, limiting our ability to infer relationships.

### 4.2 Red Squirrels

Red squirrels did not follow our expectations relative to human modification, natural disturbance, and gray squirrel presence, but did for forest composition. We found that red squirrels were less likely to be extirpated with increasing human modification, contrary to our expectations (Larson et al. 2024). We may not have found that red squirrel extirpation probability increases with increasing human modification for several reasons. Due to the design of the camera trapping survey, which excluded the most highly urbanized areas, we did not have data to evaluate the relationship in those areas. Gray squirrels may exclude red squirrels in urban areas if they exhaust urban hardwood mast resources (Larson et al. 2024). Rhode Island is highly developed but also has a high cover of forest, and areas of moderate development may have sufficient tree cover to support red squirrels and gray squirrels simultaneously. Similarly, spongy moth damage did not influence colonization or extirpation, likely because spongy moths do not prefer conifers (Dumarevskaya and Parent 2025). While we found no support that gray squirrel presence affected the initial occupancy (Moore and Swihart 2005), sites occupied by both squirrel species had lower detection probability, perhaps because red squirrel territoriality reduced abundance (Larson et al. 2024; Steele 1998). Our results confirm that red squirrel colonization probabilities increase with increasing coniferous forest cover in areas affected by timber harvest and insect damage (Johnson et al. 2015; McDermott 2021; Patterson and Malcolm 2010).

Importantly, seasonal variation impacted red squirrel occupancy dynamics. Red squirrel populations fluctuate based on interannual cone production (Dantzer et al. 2012; Gurnell 1984; Johnson et al. 2015) and their foraging and caching behavior (Steele 1998). Therefore, food availability, particularly during winter, could be driving seasonal variation in red squirrel dynamics we observed. Overall, red squirrel occupancy appears to be stable in Rhode Island, but with a slight decline in occupancy during winter and the shift towards more urban areas.

### 4.3 Cottontails

While our top model suggested that seasonality, food availability, spongy moth damage, land cover class, and human land modification would influence cottontail dynamics, few of our predictions were supported. We found no support that cottontails favored any particular land cover class, even though they are known to inhabit younger successional areas (Bischoff et al. 2025; Boland et al. 2025) and shrub habitat (Bischoff et al. 2025; Cheeseman et al. 2019) with low canopy closure (Eline et al. 2023). We may not have found a strong relationship between cottontail occupancy and any particular land cover class because they may be relatively generalist in nature and opportunistic, allowing them to take advantage of food and shelter resources in many land cover types (Hayes Hursh et al. 2023). Moreover, cottontails were less likely to colonize areas with high net primary productivity, perhaps because net primary productivity does not capture the productivity of understory vegetation, especially in areas with early regeneration, which cottontails rely on for food and cover.

Cottontails were less likely to move into or initially occupy areas with high human modification, despite evidence that extinction risk may be lower in human‐modified habitats (Verde Arregoitia et al. 2015). Cottontails were less likely to be extirpated in severely moth‐damaged areas, suggesting that natural and human disturbances may not be analogous. Cottontails were more likely to leave areas closer to roads, perhaps related to road mortality. Our findings may also highlight a limitation of our camera trapping design, in that cameras were not placed on private property or in the most urbanized areas. Cottontails often frequent yards and smaller urban properties which were under sampled in our study, so we may be capturing cottontail dynamics on public lands and are not indicative of the full range of what drives cottontail occupancy dynamics. Furthermore, our global human modification index aggregates 13 human modifications (e.g., agriculture, infrastructure, energy) into one index, which may mask varied relationships between cottontails and the individual types of human modification.

### 4.4 Eastern Chipmunks

We found that chipmunk occupancy dynamics were strongly influenced by seasonality; however, forest recovery or precipitation were not important. Our findings support the idea that eastern chipmunks are sensitive to fine‐scale environmental variation and seasonal changes in behavior (Bowers et al. 1990). Eastern chipmunks may have lower detection rates in winter when they are likely to be in torpor (Bowers 1995) where they conserve energy by reducing above‐ground activity and relying on cached food reserves (Bowers et al. 1990). Eastern chipmunk occupancy and detectability were higher at sites with rock walls. Rock walls could function as perches that have increased horizontal visibility while being relatively unobstructed, obscuring chipmunks from predators (Kitchings and Levy 1981).

Eastern chipmunk occupancy increased especially during the winter months, and more than doubled from 2019 to 2023. The increase may partially result from deploying cameras earlier in the season during later study years, capturing chipmunks before they enter torpor. The increase may also reflect population growth driven by periodic food availability and favorable weather conditions. Eastern chipmunk populations may fluctuate in response to mast availability (Dhawan et al. 2018; Mares et al. 1976), and milder winters promoting overwinter survival (Kitchings and Levy 1981). Conversely, warming winters could disrupt torpor cycles and increase mortality (Bowers 1995).

### 4.5 Small Rodents

Our small rodent group contained all small rodents we could not identify to a species level; thus, we might expect different species responses could obscure trends in occupancy dynamics. However, we found clear occupancy trends. We found that small rodent initial occupancy was influenced by forest structure, aligning with current knowledge of regionally native small rodents, including eastern moles ( *Scalopus aquaticus* ; Ritchie and Nocera 2010), *Peromyscus* spp. (Nupp and Swihart 1998; Dueser and Shuggart 1979; Fuller and DeStefano 2003), and short‐tailed shrews ( *Blarina brevicauda* ; Dueser and Shuggart 1979; Kitchings and Levy 1981). While features at camera sites, such as rock walls, may provide nesting, foraging, and hiding locations for small rodents (Johnson and Ouimet 2016; Kitchings and Levy 1981; Paliga 2011), they did not influence initial occurrence.

Small rodent detectability was influenced by season and spongy moth damage, with higher detectability in summer and moth‐damaged areas. In summer, small rodents may be more visible to cameras if they are not in burrows or under snow cover, or as populations increase due to rapid reproduction. Spongy moth damage may increase understory vegetation which could increase small rodent detectability (Dumarevskaya and Parent 2025; Pasquarella et al. 2018). Conversely, we detected the biggest increases in rodent occupancy in winter, possibly because abundant young had not been removed from the population, or because the lack of leafy vegetation improved detectability. Lower summer precipitation may promote favorable burrowing conditions, as small rodents prefer low or moderate soil moisture levels (Huey 1941; Rhodes and Richmond 1985). High soil water potential and high precipitation may promote unfavorable soil conditions (Huey 1941; Rhodes and Richmond 1985) or lead to burrow inundation. While grouping all small rodents is not ideal for parsing species differences, general trends in community‐level responses can still be gleaned from camera bycatch.

### 4.6 Limitations

Our findings support the utility of using bycatch from pre‐existing camera trapping arrays, with a few caveats. Our initial camera trapping array was designed to target mesocarnivores (fishers, bobcats); therefore, we likely had fewer detections of smaller species (chipmunks and small rodents), and arboreal species (squirrels), which may occupy a site but not within camera view. Some species may be less detectable if vegetation or snow hides them from view, or if they undergo torpor. Small‐bodied animals, especially those with camouflaging potential (i.e., chipmunks), are more difficult to detect on camera images, and may have stronger observer variation in detectability. Further, our sampling design avoided the most highly developed areas of Rhode Island, which may have explained why we found relationships with human modification that differed from relationships observed elsewhere. While bycatch data can provide valuable insights into the dynamics of nontarget species, it is important to understand these limitations, which can inform insights and implications derived from the data and steer future research design.

## 5 Conclusions

In a time when the public perception of science is declining and science funding is being heavily curtailed (Buza et al. 2025; Olesko et al. 2025), camera trapping data, which is expensive and time consuming to generate, should be utilized to its maximum to make limited research dollars go further. Species which are widely distributed such as small rodents, squirrels, and cottontails are bycatch in many camera trapping studies targeting species but are often underrepresented in research (dos Santos et al. 2020), even though they have significant impacts on ecosystem functioning and resilience (Carey and Harrington 2001), are indicators of ecosystem health (Pearce and Venier 2005; Schmidt‐Nielsen 1984), and can be economically important (Boland and Litvaitis 2008; Kays et al. 2017). Common, widespread species at lower trophic levels drive ecosystem functioning and biodiversity patterns, and even a small decline equates to a large loss of biomass and species interactions. Our focal taxa (sciurids, lagomorphs, and rodents) occupy those lower trophic levels, often have cyclical population dynamics, and like vegetation dynamics and predators in food webs. They further can have important implications for humans: hunting opportunities, damage to crops, gardens, and houses, and as vectors of zoonotic disease and parasites. Data on nontarget species captured via pre‐existing camera trap studies can be utilized to provide new or improved insights about species that may not be assessed otherwise, ecosystem health and community functioning, and offer potential predictors to use in analyses of target species that may rely on nontarget species as prey or inform insights about human–wildlife interactions or human disease.

## Author Contributions

**Ashley M. Olah:** conceptualization (equal), data curation (equal), methodology (equal), project administration (equal), visualization (equal), writing – original draft (lead), writing – review and editing (equal). **Laken S. Ganoe:** conceptualization (equal), data curation (equal), formal analysis (lead), methodology (equal), project administration (equal), visualization (lead), writing – original draft (equal), writing – review and editing (equal). **Christopher J. Hickling:** conceptualization (equal), writing – original draft (equal), writing – review and editing (equal). **Theint Thandar Bol:** conceptualization (equal), writing – original draft (equal), writing – review and editing (equal). **Donald Ruggieri:** conceptualization (equal), writing – original draft (equal), writing – review and editing (equal). **Amy Mayer:** conceptualization (equal), data curation (equal), writing – original draft (equal), writing – review and editing (equal). **Kathleen A. Carroll:** conceptualization (equal), supervision (lead), writing – original draft (equal), writing – review and editing (equal).

## Funding

This work was supported by the US Fish and Wildlife Service (Grant F19AF01093).

## Conflicts of Interest

The authors declare no conflicts of interest.

## Data Availability

The dataset and model code for these analyses are available on Zenodo at https://doi.org/10.5281/zenodo.17260941. The detection dataset is available as Supporting Information to the paper (Mayer et al. 2025) and is also available on Zenodo at https://doi.org/10.5281/zenodo.10610602. The raw images that were used to compile the dataset are owned by the University of Rhode Island Department of Natural Resources Science. Qualified researchers may request the raw images by contacting the University of Rhode Island Department of Natural Resources Science Quest/Gerber Lab Manager (current email: agottfried@uri.edu) and requesting Rhode Island camera survey data from 2020 through 2023.

## Appendix

**TABLE A1:** Predictions about gray squirrel, red squirrel, eastern chipmunk, cottontails, and rodent responses to predictor variables based on specific ecology, whether the predictions were supported in models, and associated references.

Species	Variable	Ecology	Predicted response	Supported	Citations
Gray Squirrel	Season	In summer gray squirrels are more active and male home ranges expand, but more vegetation in summer may obscure species from cameras	Greater detectability and colonization in summer with increases in activity and home range sizes. Greater extirpation in winter with decreases in activity and shrinking home ranges	Yes	Bland (1977); Larson et al. (2024); Thompson (1977)
Gray Squirrel	Precip_S	More precipitation in previous season may lead to greater food availability in the next season	Gray squirrel colonization will be greater when precipitation the previous season is greater because food is abundant. Extirpation will be greater when precipitation in the previous season is low because food resources are low		Allen et al. (2018, 201); Wion et al. (2021)
Gray Squirrel	TSD_S	Spongy moth damage defoliates trees, reducing canopy cover and mast resources. Spongy moth damage may promote the creation of nest cavities in trees which gray squirrels prefer, but the loss of canopy cover may negatively affect gray squirrel use of moth damaged areas	Gray squirrels may be less likely to colonize and more likely to leave areas over time, in conjunction with spongy moth damage		Twery (1990); Koprowski (1994); Fuller and DeStefano (2003)
Gray Squirrel	pct_conifer	Gray squirrels may prefer deciduous or mixed deciduous‐coniferous forests, but will use coniferous forests; coniferous forest may be a barrier to movement	Gray squirrel occupancy, detection, and colonization will be lower in coniferous forests		Bertolino et al. (2008); Fischer and Holler (1991); Gonzales (2005); Koprowski (1994); Koprowski et al. (2016); Riege (1991)
Gray Squirrel	pct_mixed	Gray squirrels may prefer deciduous or mixed deciduous‐coniferous forests, but will use coniferous forests; coniferous forest may be a barrier to movement	Gray squirrel occupancy and detection will be higher in mixed forests. Gray squirrels may move in and out of mixed forests to utilize deciduous trees		Bertolino et al. (2008); Fischer and Holler (1991); Gonzales (2005); Koprowski (1994); Koprowski et al. (2016); Riege (1991)
Gray Squirrel	Percent coniferous and mixed forest (pct_conmix)	Gray squirrels may prefer deciduous or mixed deciduous‐coniferous forests, but will use coniferous forests; coniferous forest may be a barrier to movement. Gray squirrels need contiguous forests	Gray squirrel occupancy will be higher in areas with more forest around camera sites		Koprowski (1994); Koprowski et al. (2016); Moore and Swihart (2010); Nixon et al. (1978)
Gray Squirrel	lc_class	Gray squirrels need forests and avoid open areas	Gray squirrel occupancy and detections will be greatest in forest land cover classes. They will not colonize other land cover classes		Bertolino et al. (2008); Koprowski (2005); Nixon et al. (1978); Tounzen et al. (2012)
Gray Squirrel	contPCA	Gray squirrels may prefer greater canopy cover and mature trees. Tree maturity may be related to increased canopy cover. Gray squirrels avoid early successional habitats	Gray squirrel occupancy will be greater in mature forests (those with greater canopy cover)	Yes	Brown and Batzli (1984); Fuller and DeStefano (2003); Riege (1991); Williams (2011)
Gray Squirrel	avg_dist_water	Water is an important resource for organisms. Permanent water sources may be important particularly in dry areas or periods of the year	Gray squirrel occupancy and detections may be greater nearer water		Johnston et al. (2020)
Gray Squirrel	avg_dist_roads	Roads are sources of habitat fragmentation, mortality and other human disturbance	Gray squirrel detectability may be lower near roads		Engel et al. (2020); Kay et al. (2023)
Gray Squirrel	avg_npp	Net primary productivity is related to food availability, with areas of higher net primary productivity likely to have more abundant food. Primary productivity may interact with precipitation to drive mast years	Gray squirrel colonization will be greater when primary productivity is greater because food is abundant. Extirpation will be greater when primary productivity is low because food resources are low	Yes	Allen et al. (2018); Garcia et al. (2021); Mund et al. (2020); Wion et al. (2021)
Gray Squirrel	gHM	Human modification alters species' habitats, behavior, space and resource use, density, and occupancy. Gray squirrels seem to adapt well to human modification	Gray squirrel occupancy and detectability will be greater in areas with more human modification	Yes	Engel et al. (2020); Gonzales (2005); Tranquillo et al. (2024)
Gray Squirrel	temp_winter	Winter severity may negatively impact species. Food resources are reduced and thermoregulation costs may increase. Gray squirrel movements may be reduced when winter temperatures increase	Gray squirrel detectability may be lower with lower winter temperatures		Bland (1977)
Gray Squirrel	maj_moth	Spongy moth damage defoliates trees, reducing canopy cover and mast resources. Gray squirrels may have already moved out of areas with severe spongy moth damage. Gray squirrels do not use early successional habitat	Gray squirrel initial site occupancy will be lower in heavily moth damaged areas. They will be less likely to colonize areas with severe moth damage (low food availability, canopy cover), and more likely to move out of areas of severe moth damage		Fuller and DeStefano (2003); Koprowski (1994); Twery (1990)
Gray Squirrel	moth_pres	Spongy moth damage defoliates trees, reducing canopy cover and mast resources. Spongy moth damage may promote the creation of nest cavities in trees which gray squirrels prefer, but the loss of canopy cover may negatively affect gray squirrel use of moth damaged areas	Gray squirrels will be less likely to colonize areas with spongy moth damage, and more likely to move out of areas with spongy moth damage		Fuller and DeStefano (2003); Koprowski (1994); Twery (1990)
Red Squirrel	Season	Vegetation in summer may obscure cameras from detecting red squirrels. Winter food caches are abandoned in summer. Red squirrels have different territories in different seasons. Deciduous areas are more important in winter	Lower detectability in summer. Colonization of deciduous areas may increase in summer, and extirpation of deciduous areas may increase in winter	Yes	Kemp and Keith (1970)
Red Squirrel	SeasonYear	Red squirrel site occupancy varies with inter‐annual cone production	Site colonization and extirpation vary between years	Yes	Dantzer et al. (2012); Gurnell (1984); Johnson et al. (2015)
Red Squirrel	Precip_S	Heavy rain and snow reduce activity levels. Cone crops in late summer are negatively related to rainfall in the previous summer	Detectability will be lower with higher precipitation. Colonization of sites will increase with lower precipitation in the previous summer. Extirpation of sites will increase with higher precipitation in the previous summer		Kemp and Keith (1970); Layne (1954)
Red Squirrel	TSD_S	Spongy moth damage defoliates trees. Red squirrel detectability is greater in regenerating forests but red squirrels also avoid regenerating forests	Red squirrels may be less likely to colonize and more likely to leave areas over time, in conjunction with spongy moth damage		Johnson et al. (2015); McDermott (2021)
Red Squirrel	Gray Squirrel Presence	Red squirrels rarely co‐occur with gray squirrels, and if they do, they occur at lower abundances	Where gray squirrels are present, red squirrel detectability will be lower, colonization will be lower, and extirpation will be greater	Yes	Larson et al. (2024); Moore and Swihart (2010)
Red Squirrel	pct_conifer	Red squirrels are associated with mature coniferous forests	Initial site occupancy and colonization will be greater with greater cover of coniferous forests	Yes	Johnson et al. (2015); McDermott (2021); Patterson and Malcolm (2010)
Red Squirrel	pct_mixed	Red squirrels are associated with mature coniferous forests, but deciduous areas may be important in winter for juveniles	Initial site occupancy may be greater in areas with greater mixed forest cover. Colonization may be higher and extirpation lower in sites of greater mixed forest cover		Johnson et al. (2015); Kemp and Keith (1970); McDermott (2021); Patterson and Malcolm (2010)
Red Squirrel	pct_conmix	Red squirrels are associated with mature coniferous forests, but deciduous areas may be important in winter for juveniles	Initial site occupancy may be greater in areas with greater cover of mixed and coniferous forest. Colonization may be higher and extirpation lower in sites of greater cover of mixed and coniferous forest		Johnson et al. (2015); Kemp and Keith (1970); McDermott (2021); Patterson and Malcolm (2010)
Red Squirrel	contPCA	Red squirrels are associated with mature coniferous and deciduous forest and have limited dispersal in young forests. Tree maturity may be related to increased canopy cover	Red squirrel occupancy and detections will be greatest in mature forests (those with greater canopy cover). They will not colonize other land cover classes. Detectability and colonization will be lower in young forests, and extirpation will be higher		Johnson et al. (2015); Kemp and Keith (1970); Larsen (2009); McDermott (2021); Patterson and Malcolm (2010)
Red Squirrel	avg_dist_water	Red squirrels avoid wet areas	Red squirrels detectability and colonization will be lower closer to water, and extirpation may be higher		McDermott (2021)
Red Squirrel	avg_dist_roads	Roads are sources of habitat fragmentation, mortality, and other human disturbance	Red squirrel initial site occupancy, detectability and colonization will be lower nearer roads, and extirpation of sites will be higher		Chen and Koprowski (2015); Hill et al. (2021)
Red Squirrel	gHM	Human modification alters species' habitats, behavior, space and resource use, density, and occupancy. Red squirrels occur at low levels in areas of high urban intensity	Red squirrel initial occupancy and detectability will be lower in areas of greater human modification	Yes	Larson et al. (2024); Tranquillo et al. (2024)
Red Squirrel	maj_moth	Spongy moth damage defoliates trees, reducing canopy cover and mast resources, but may increase snag density. Red squirrels need high snag density but also live trees, and avoid regenerating early successional forests	Red squirrel initial site occupancy will be lower in heavily moth damaged areas, but detectability may be greater. Colonization may be lower with severe moth damage as red squirrels avoid regenerating forests, but extirpation may be lower with increasing moth damage if red squirrels respond to increasing snag density		Johnson et al. (2015); McDermott (2021)
Eastern Chipmunk	Season	Vegetation in summer may obscure cameras from detecting eastern chipmunks. Eastern chipmunks enter torpor in winter, reduce above ground activity, and rely on cached food reserves	Lower detectability in winter than in summer	Yes	Bowers (1995); Bowers et al. (1990)
Eastern Chipmunk	Precip_S	Home range sizes increase in periods of drought and with lack of food. Droughts lead to population declines. Precipitation in the previous season may lead to increased food abundance and colonization	Higher precipitation in the previous season increases colonization probabilities, and reduces extirpation and detection probabilities		Bowers et al. (1990); Dhawan et al. (2018); Mares et al. (1976)
Eastern Chipmunk	TSD_S	Spongy moth damage defoliates trees, reducing food resources	Eastern chipmunks may be less likely to colonize and more likely to leave areas over time, in conjunction with spongy moth damage	Yes	Mares et al. (1976)
Eastern Chipmunk	contPCA	Eastern chipmunks are associated with woodland, and may favor second growth, but presence is lower in areas of abundant herbaceous vegetation	Eastern Chipmunk occupancy will be lower closer to young forests		Kitchings and Levy (1981)
Eastern Chipmunk	avg_dist_water	Eastern chipmunks avoid swampy areas	Eastern chipmunk detectability and colonization may increase and extirpation may decrease with distance from water sources	Yes	Kitchings and Levy (1981)
Eastern Chipmunk	avg_npp	Net primary productivity is related to food availability, with areas of higher net primary productivity likely to have more abundant food. Primary productivity may interact with precipitation to drive mast years	Chipmunk colonization will be greater when primary productivity is greater because food is abundant. Extirpation will be greater when primary productivity is low because food resources are low		Allen et al. (2018); Garcia et al. (2021); Mund et al. (2020); Wion et al. (2021)
Eastern Chipmunk	gHM	Human modification alters species' habitats, behavior, space and resource use, density, and occupancy. Chipmunks forage more frequently in urban habitat. In fragmented corridors chipmunks locomote and forage more, and pause more frequently	Chipmunks detectability and extirpation will be higher, but occupancy and colonization will be lower in human modified areas		Larson et al. (2024); Mahan and Yahner (1999); Tranquillo et al. (2024)
Eastern Chipmunk	soilPCA	Sandy soils are easier for chipmunks to dig burrows. When clay is encountered, burrowing ends	Occupancy and colonization are greater in areas with higher soil PCA values (greater sand content)		Panuska and Wade (1956)
Eastern Chipmunk	maj_moth	Spongy moth damage defoliates trees, reducing food resources	Eastern chipmunk initial site occupancy will be lower in heavily moth damaged areas. They will be less likely to colonize areas with severe moth damage (low food availability), and more likely to move out of areas of severe moth damage	Yes	Mares et al. (1976)
Eastern Chipmunk	features	Eastern chipmunks may use rock walls, stumps, and logs as burrows, runways, and vantage points to observe predators. They don't venture far from burrows	Eastern chipmunk occupancy and detectability will be greater near features such as logs, stumps, or rock walls	Yes	Burrows (1995); Kitchings and Levy (1981); Kolowski and Forrester (2017)
Eastern Chipmunk	rock_wall	Eastern chipmunks may use rock walls, stumps, and logs as burrows, runways, and vantage points to observe predators. They don't venture far from burrows	Eastern chipmunk occupancy and detectability will be greater near features such as logs, stumps, or rock walls		Bowers (1995); Kitchings and Levy (1981); Kolowski and Forrester (2017)
Cottontail	Season	Vegetation in summer may obscure cameras from detecting cottontails. Cottontails reproduce rapidly in summer and juveniles disperse. Cottontails have short lifespans, and in winter many have died or will die	Detectability is lower in summer, while colonization of new sites is high. In winter, extirpation of sites is higher than in summer	Yes	Chapman et al. (1977); Moll, Ortiz‐Calo, et al. (2020); Trethewey and Verts (1971)
Cottontail	Precip_S	Precipitation in the previous season may lead to increased food abundance and colonization	Higher precipitation in the previous season increases colonization probabilities, and reduces extirpation and detection probabilities		Allen et al. (2018); Wion et al. (2021)
Cottontail	TSD_S	Cottontails select for early successional habitats, with less canopy closure. Spongy moth damage defoliates trees and reduces canopy cover, resetting succession and creating preferred habitats	Colonization increases over time in areas defoliated by spongy moths		Bischoff et al. (2025); Cheeseman et al. (2019); Eline et al. (2023); Fuller and DeStefano (2003)
Cottontail	lc_class	Cottontails select for early successional habitats, with less canopy closure, such as shrubland, young forest, and understory vegetation	Initial occupancy is greater in early successional habitats		Bischoff et al. (2025); Cheeseman et al. (2019); Eline et al. (2023); Fuller and DeStefano (2003)
Cottontail	contPCA	Cottontails select for early successional habitats	Initial occupancy is greater in young forests		Eline et al. (2023); Fuller and DeStefano (2003)
Cottontail	avg_dist_roads	Changes in plant communities along roads produce high quality forage, produce cover during periods of heavy snow, and mowed vegetation may facilitate movement between habitat patches. Fencing along roads may create a predator‐free zone between the road and the fence. Roads also represent sources of mortality	Detectability, occupancy, and colonization are greater nearer roads	Yes	Hill et al. (2021)
Cottontail	avg_npp	Net primary productivity is related to food availability, with areas of higher net primary productivity likely to have more abundant food. Primary productivity may interact with precipitation to drive vegetation productivity	Colonization will be greater when primary productivity is greater because food is abundant. Extirpation will be greater when primary productivity is low because food resources are low	Yes	Allen et al. (2018); Garcia et al. (2021); Mund et al. (2020); Wion et al. (2021)
Cottontail	gHM	Human modification alters species' habitats, behavior, space and resource use, density, and occupancy	Occupancy and detectability are greater with increasing human modification, while extirpation is reduced	Yes	Fidino et al. (2021); Moll, Cepek, et al. (2020); Verde Arregoitia et al. (2015)
Cottontail	maj_moth	Cottontails select for early successional habitats, with less canopy closure, such as shrubland, young forest, and understory vegetation	Initial occupancy and colonization are greater in areas with more moth damage; extirpation is lower with increasing moth damage	Yes	Bischoff et al. (2025); Cheeseman et al. (2019); Eline et al. (2023); Fuller and DeStefano (2003)
Cottontail	moth_pres	Cottontails select for early successional habitats, with less canopy closure, such as shrubland, young forest, and understory vegetation	Initial occupancy and colonization are greater with moth presence while extirpation is lower		Bischoff et al. (2025); Cheeseman et al. (2019); Eline et al. (2023); Fuller and DeStefano (2003)
Cottontail	rockwall	Cottontails may use rock walls as denning or resting sites. Rock walls may impede movements, direct cottontail movements, and act as travel corridors	Detectability increases with the presence of Rockwall within the camera site	Yes	Boland et al. (2025); Paliga (2011)
Rodents	Season	Vegetation in summer may obscure cameras from detecting rodents. Different rodents may be active at different times of year	Detectability is lower in summer. Occupancy, colonization and extirpation differ between seasons	Yes	Moll, Ortiz‐Calo, et al. (2020); Scott et al. (2022)
Rodents	Precip_S	Precipitation in the previous season may lead to increased food abundance and colonization. Periods of drought reduce population growth rates	Higher precipitation in the previous season increases colonization probabilities and reduces extirpation and detection probabilities	Yes—interaction with water potential	Dhawan et al. (2018); Scott et al. (2022)
Rodents	TSD_S	Rodent species favor forest cover, others favor open woodlands, and others avoid early successional habitats. Spongy moth damage defoliates trees and reduces canopy cover, resetting succession	Time since defoliation interacts with damage to reduce detectability and occupancy	Yes	Fuller and DeStefano (2003); Moore and Swihart (2010)
Rodents	lc_class	Rodent species favor forest cover, others favor open woodlands, and others avoid early successional habitats	Occupancy will be greater in forest land cover classes		Fuller and DeStefano (2003); Nupp and Swihart (1998); Ritchie and Nocera (2010)
Rodents	contPCA	Some rodents may favor mature forests	Occupancy will be greater in mature forests than younger forests	Yes	Fuller and DeStefano (2003); Nupp and Swihart (1998); Ritchie and Nocera (2010)
Rodents	avg_dist_roads	Roads allow for rapid movement to burrows when threatened by predators. Vegetation along roads provides cover and facilitate movement between habitat patches. The zone between roads and roadside fences may be predator‐free. Roadsides may have favorable soil conditions for burrows. Roads also represent a source of mortality	Initial occupancy, detectability, and colonization are higher near roads. Extirpation is lower near roads		Hill et al. (2021)
Rodents	avg_npp	Net primary productivity is related to food availability, with areas of higher net primary productivity likely to have more abundant food. Primary productivity may interact with precipitation to drive vegetation productivity. Higher primary productivity may be related to greater vegetation cover	Initial occupancy is higher in areas of greater primary productivity		Allen et al. (2018); Garcia et al. (2021); Kitchings and Levy (1981); Mund et al. (2020); Wion et al. (2021)
Rodents	gHM	Human modification alters species' habitats, behavior, space and resource use, density, and occupancy. Human modification alters community compositions. Species likely have different responses to human modification	Occupancy, detectability, colonization, and extirpation may increase or decrease with urbanization		Larson and Sander (2025); Moore and Swihart (2010); Persons and Eason (2017); Tranquillo et al. (2024)
Rodents	soilPCA	The proportion of sand, silt, and clay influence the ability of rodents to create burrows. Sandy soil is easier to dig than soils with high clay content	Occupancy and colonization are greater in areas with higher soil PCA values (greater sand content)		Laundré (1989); Laundré and Reynolds (1993); Ritchie and Nocera (2010)
Rodents	watpot	Intermediate or high soil moisture may facilitate burrowing. Soil near water or areas with high water tables may cause burrow inundation. Precipitation combined with high soil moisture may cause inundation	Occupancy will be higher in areas of intermediate soil water potential. Extirpation will be higher in areas with high water potential and high precipitation	Yes—Interaction with precipitation	Huey (1941); Rhodes and Richmond (1985)
Rodents	avg_bulkden	Soil texture influences rodent burrow length, diameter, and volume, with loose soils being easier to burrow into than harder soils. Soil hardness discourages burrowing but increases use of pre‐existing burrows	Occupancy is greater in areas with loose soils		Kitchings and Levy (1981); Laundré (1989); Laundré and Reynolds (1993); Meliyo et al. (2015)
Rodents	maj_moth	Rodent species favor forest cover, others favor open woodlands, and others avoid early successional habitats. Spongy moth damage defoliates trees and reduces canopy cover, resetting succession	Occupancy and detectability are reduced in severely damaged areas, in conjunction with time since defoliation		Fuller and DeStefano (2003); Moore and Swihart (2010)
Rodents	moth_pres	Rodent species favor forest cover, others favor open woodlands, and others avoid early successional habitats. Spongy moth damage defoliates trees and reduces canopy cover, resetting succession	Occupancy and detectability are reduced in areas where moths are present	Yes	Fuller and DeStefano (2003); Moore and Swihart (2010)
Rodents	features	Features such as woody debris, rock walls, or rock piles may provide nesting, foraging, and hiding locations for rodents	Initial occupancy and detectability are greater at sites with local features		Johnson and Ouimet (2016); Kitchings and Levy (1981); Paliga (2011)
Rodents	features2	Features such as woody debris, rock walls, or rock piles may provide nesting, foraging, and hiding locations for rodents	Initial occupancy and detectability are greater at sites with local features		Johnson and Ouimet (2016); Kitchings and Levy (1981); Paliga (2011)

**TABLE A2:** Habitat variables used in occupancy models of gray squirrel, red squirrel, eastern chipmunk, cottontail, and rodents in Rhode Island, and associated descriptions and sources.

Variable name		Description	Format	Resolution	Seasonal or site	Source
	Season	Summer/winter	Factor (2 levels)	n/a	Seasonal	
	SeasonYear	Summer/winter by year	Factor (8 levels)	n/a	Seasonal	
	Precip_S	Average seasonal precipitation from the previous season	Continuous	27m^2^	Seasonal	NOAA (2025)
	TSD_S	Time Since Disturbance, i.e., time since initial spongy moth outbreak and defoliation	Continuous	n/a	Seasonal	Pasquarella (2018); Pasquarella et al. (2017)
	gsquirrel	Were gray squirrels detected at site in each season (present, absent)	Factor (2 levels)	n/a	Seasonal	
	pct_conifer	Percent of 50 or 100 m buffer around site centroid that is coniferous forest	Continuous	1km^2^	Site	RIGIS (2021)
	pct_mixed	Percent of 50 or 100 m buffer around site centroid that is mixed forest	Continuous	1km^2^	Site	RIGIS (2021)
	pct_conmix	Percent of 50 or 100 m buffer around site centroid that is either mixed or coniferous	Continuous	1km^2^	Site	RIGIS (2021)
	lc_class	Landcover class (forest, pasture/grassland, shrub/scrub) at site centroid	Factor (3 levels)	27m^2^	Site	RIGIS (2021)
contPCA (Principal component consisting of lc_class variety, avg_canopy, and avg_dist_yf. Positive values = more landscape heterogeneity and more young forest, Negative values = older, more contiguous forests)	lc_class_variety	The number of land cover classes within a 50 or 100 m buffer around site centroid	Continuous	n/a	Site	RIGIS (2021)
	avg_canopy	Mean canopy height in 50 or 100 m buffer around site centroid	Continuous	1 km^2^	Site	
	avg_dist_yf	Mean distance within 50 or 100 m buffer around site centroid to nearest young forest	Continuous	27 m^2^	Site	RIGIS (2023b)
	avg_dist_water	Mean distance within 50 or 100 m buffer around site centroid to nearest fresh water	Continuous	27 m^2^	Site	RIGIS (2001, 2023a)
	avg_dist_roads	Mean distance within 50 or 100 m buffer around site centroid to nearest road	Continuous	27 m^2^	Site	RIGIS (2021)
	avg_npp	Mean net primary productivity (measure of biomass availability to the next trophic level) within 50 or 100 m buffer around site centroid	Continuous	27 m^2^	Site	Robinson et al. (2018)
	gHM	Human modification index at site centroid (0–1 scale; 1 = most modified)	Continuous	1 km^2^	Site	Kennedy et al. (2019); Theobald et al. (2025)
soilPCA (Principal component of avg_silt, avg_sand, avg_clay. More negative values = higher clay and silt content, more positive values = higher sand content)	avg_silt	Mean silt soil composition in 50 or 100 m buffer around site centroid	Continuous	5 m^2^	Site	RIGIS (2020); Soil Survey Staff (n.d.)
	avg_sand	Mean sand soil composition in 50 or 100 m buffer around site centroid	Continuous	5 m^2^	Site	RIGIS (2020); Soil Survey Staff (n.d.)
	avg_clay	Mean clay soil composition in 50 or 100 m buffer around site centroid	Continuous	5 m^2^	Site	RIGIS (2020); Soil Survey Staff (n.d.)
	avg_bulkden	Mean bulk density (Db 0.33 bar H20) of soil within 50 or 100 m buffer around site centroid	Continuous	5 m^2^	Site	RIGIS (2020); Soil Survey Staff (n.d.)
	avg_watpot	Mean water potential (0.33 bar H20) of soil within 50 or 100 m buffer around site centroid	Continuous	5 m^2^	Site	RIGIS (2020); Soil Survey Staff (n.d.)
	maj_moth	Majority value of spongy moth damage (0–3 classification) in 50 or 100 m of site centroid	Continuous	30 m^2^	Site	Pasquarella (2018); Pasquarella et al. (2017)
	med_moth	Median value of moth damage (0–3 classification) in 50 or 100 m of site centroid	Continuous	n/a	Site	Pasquarella (2018); Pasquarella et al. (2017)
	moth_pres	Presence or absence of spongy moth damage	Factor (2 levels)	30 m^2^	Site	Pasquarella (2018); Pasquarella et al. (2017)
	features	What feature is prominent in front of camera (rock wall, woody, mixed, rocky, none). Both cameras combined	Factor (5 levels)	n/a	Site	
	features2	What feature is prominent in front of camera reclassified into less parameters (rock wall, mixed, none). Both cameras combined	Factor (3 levels)	n/a	Site	
	rockwall	Rock wall presence (present/absent) in front of camera. Both cameras combined	Factor (2 levels)	n/a	Site	
