# Correction to “Assessment of Nontarget Small Mammal Occupancy Using Broadly Designed Camera Arrays”

**DOI:** 10.1002/ece3.74072

**Published:** 2026-07-20

Olah, A. M. , L. S. Ganoe , C. J. Hickling , et al. 2026. “Assessment of Nontarget Small Mammal Occupancy Using Broadly Designed Camera Arrays” Ecology and Evolution 16, no. 6: e73834. 10.1002/ece3.73834.42328476 PMC13282166

Figures 5, 6, 9, and 10 contain labelling errors and incorrect species images. Figures 5 and 6 are labelled as showing results for red squirrels and include images of red squirrels; however, they actually present results for eastern chipmunks. Conversely, Figures 9 and 10 are labelled as showing results for eastern chipmunks and include images of chipmunks, but they in fact present results for red squirrels.

These errors also affect figure citations within the Results section. In Section 3.2 (red squirrels), Figure 5 is incorrectly cited in place of Figure 9, and Figure 6 is cited in place of Figure 10. Similarly, in Section 3.4 (eastern chipmunks), Figure 9 is incorrectly cited in place of Figure 5, and Figure 10 is cited in place of Figure 6.

We apologize for this error.

